# Assessing the relationship between gut microbiota and irritable bowel syndrome: a two-sample Mendelian randomization analysis

**DOI:** 10.1186/s12876-023-02791-7

**Published:** 2023-05-12

**Authors:** Bin Liu, Ding Ye, Hong Yang, Jie Song, Xiaohui Sun, Zhixing He, Yingying Mao, Guifeng Hao

**Affiliations:** 1 Department of Epidemiology, School of Public Health, Zhejiang Chinese Medical University, Hangzhou, 310053 China; 2 Institute of Basic Research in Clinical Medicine, School of Basic Medical Science, Zhejiang Chinese Medical University, Hangzhou, 310053 China; 3 Center for General Practice Medicine, Department of Rheumatology and Immunology, Zhejiang Provincial People’s Hospital (Affiliated People’s Hospital, Hangzhou Medical College), Hangzhou, 310014 China

**Keywords:** Irritable bowel syndrome, Gut microbiota, Mendelian randomization, Single nucleotide polymorphism

## Abstract

**Background:**

Growing evidence has suggested that gut microbiota is closely related to the risk of irritable bowel syndrome (IBS), but whether there is a causal effect remains unknown. We adopted a Mendelian randomization (MR) approach to evaluate the potential causal relationships between gut microbiota and the risk of IBS.

**Methods:**

Genetic instrumental variables for gut microbiota were identified from a genome-wide association study (GWAS) of 18,340 participants. Summary statistics of IBS were drawn from a GWAS including 53,400 cases and 433,201 controls. We used the inverse-variance weighted (IVW) method as the primary analysis. To test the robustness of our results, we further performed the weighted-median method, MR-Egger regression, and MR pleiotropy residual sum and outlier test. Finally, reverse MR analysis was performed to evaluate the possibility of reverse causation.

**Results:**

We identified suggestive associations between three bacterial traits and the risk of IBS (odds ratio (OR): 1.08; 95% confidence interval (CI): 1.02, 1.15; *p* = 0.011 for phylum Actinobacteria; OR: 0.95; 95% CI: 0.91, 1.00; *p* = 0.030 for genus *Eisenbergiella* and OR: 1.10; 95% CI: 1.03, 1.18; *p* = 0.005 for genus *Flavonifractor*). The results of sensitivity analyses for these bacterial traits were consistent. We did not find statistically significant associations between IBS and these three bacterial traits in the reverse MR analysis.

**Conclusions:**

Our systematic analyses provide evidence to support a potential causal relationship between several gut microbiota taxa and the risk of IBS. More studies are required to show how the gut microbiota affects the development of IBS.

**Supplementary Information:**

The online version contains supplementary material available at 10.1186/s12876-023-02791-7.

## Background

Irritable bowel syndrome (IBS) is a chronic functional gastrointestinal disorder that affects 11% of the world’s population [1]. IBS affects more women than men, and adults younger than 50 years of age compared with older ones [2]. The main symptoms of IBS include abdominal pain, changes in defecation habits and/or fecal condition, abdominal distension, and discomfort [3]. IBS imposes a large burden on patients, impairing health-related quality of life and work productively [4]. Traditional therapeutic approaches for IBS, including dietary changes and antibiotic therapy, may not obtain satisfactory outcomes since most of them are treating symptoms. Recently, the prevalence of IBS has been rising all over the world, mainly due to anxiety and stress [5].

The pathophysiological mechanisms underlying IBS are multifactorial and have been poorly understood. A heritable component of IBS is long recognized in family and twin studies [6]. Evidence is now accumulating that genetic risk in IBS spans from complex polygenic conditions with combinations of common variants to cases with rare single gene abnormalities [7, 8]. Recent studies have shown that gut microbiota may be related to the pathogenesis of IBS [9–11]. Treatment with antibiotics or fecal microbiota transplantation relieves global IBS symptoms without causing constipation, suggesting a direct relationship between gut microbiota and IBS [12, 13]. A recent systematic review has pointed out that alterations of gut microbiota exist in patients with IBS, which might exert a pivotal role in the development of IBS [14].

Although gut microbiota has been related with IBS, the causal nature is elusive. Mendelian randomization (MR) analysis is a statistical approach that aims to infer potentially causal relationships from observational association results [15]. MR uses genetic variants associated with exposure as a surrogate for exposure to assess the relationship between the surrogate and the outcome [16]. In recent years, MR analysis has been applied to assess the potential causal relationships between gut microbiota and disease-risking genes [17–19]. So far, there is an urgent need to investigate the potential causal relationship between gut microbiota and the risk of IBS.

In the present study, in order to explore the potential causal relationship between gut microbiota and IBS, and to identify specific pathogenic bacteria taxa, we conducted a two-sample MR study based on genome-wide association study (GWAS) summary data.

## Methods

### Outcome data sources

**Fig. 1:**
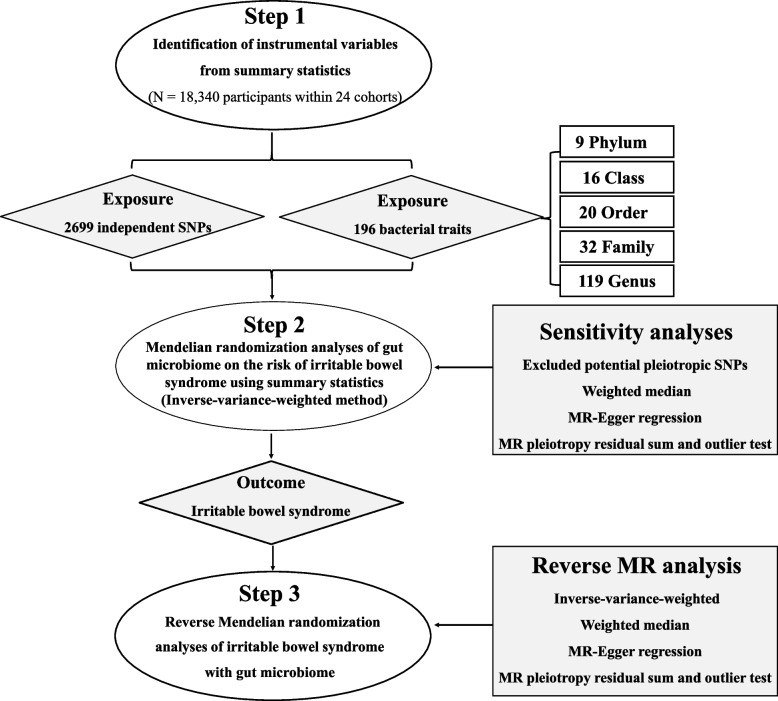
The study design of the associations of gut microbiota and irritable bowel syndrome. Abbreviations: MR, Mendelian randomization; SNP, single nucleotide polymorphism

The overall design of the present study is presented in Fig. 1. Briefly, genetic summary statistics for IBS were generated from a GWAS including 53,400 cases and 433,201 controls of European ancestry, which combined data from UK Biobank and Bellygenes initiative [20]. All patients with IBS satisfied at least one of the following four conditions: 1) satisfied the Rome III symptom criteria for IBS diagnosis and did not have other explanations for their symptoms; 2) they admitted that they have been diagnosed with IBS; 3) they self-reported they met IBS diagnosis; and 4) linked hospital episode statistics indicating inpatient or day-case admission with clinician diagnosis of IBS entered as ICD-10 diagnosis [20].

The summary statistics for human gut microbiome we used in this study were obtained from the most recent GWAS meta-analysis, which included 18,340 participants from 24 cohorts [21]. Detailed of the study has been described elsewhere [21]. Briefly, the study coordinated 16S rRNA gene sequencing profiles and genotyping data from cohorts from the USA, Canada, Germany, Denmark, the Netherlands, Belgium, Sweden, Finland, the UK and so on, and performed the association analyses with adjustment for age, sex, technical covariates, and genetic principal components [21]. As the present study was based on public summary data, no additional ethics approval or consent to participate was required. The details of the data sources in this MR study are shown in Table 1.

**Table 1:** Details of the genome-wide association studies and datasets used in our analyses

Exposure or outcome	Sample size	Ancestry	Links for data download	PMID
Human gut microbiome	18,340 participants	Mixed	https://mibiogen.gcc.rug.nl	33462485
Irritable bowel syndrome	53,400 cases, 433,201 controls	European ancestry	http://ftp.ebi.ac.uk/pub/databases/gwas/summary_statistics/GCST90016001-GCST90017000/GCST90016564/	34741163

### Selection of instrumental variables

We first removed 15 bacterial traits without specific name, leaving 196 bacterial traits, including 9 Phylum, 16 Class, 20 Order, 32 Family and 119 Genus. Then, we selected the instrumental variables (IVs) at *p* < 1.0 × 10^–5^. In order to obtain IVs from independent loci, we set the linkage disequilibrium (LD) threshold at R^2^ < 0.001 and clumping distance = 10,000 kb in 1000 Genomes EUR data using “TwoSampleMR” packages. Single nucleotide polymorphisms (SNPs) with the lowest *p*-value for the associated trait were retained for clumping with 196 bacterial traits. A total of 2699 independent SNPs were found to be associated with 196 bacterial traits. In the reverse MR analysis, we selected IVs associated with IBS at a stricter threshold (*p* < 5 × 10^–8^) which has been described in the previous study (Table 2) [20]. After extracted relevant information such as effect allele, effect size including β-value, standard error and *P*-value for each SNP, we calculated the proportion of variation explained (R^2^) and F-statistics to quantify the instrument strength, with the following equation: *R*^2^ = 2 × MAF × (1 − MAF) × β^2^, F = R^2^ (n-k-1) / k(1-R^2^), where "MAF" is the minor allele frequency of SNPs used as IVs, "n" is the sample size, and "k" is the number of IVs employed [22, 23].

**Table 2:** Characteristics of the genetic variants associated with the risk of IBS

SNP	Chr	Position	Effect allele	Beta	SE	*p*-value
rs1248825	3	84,993,411	A	0.044	0.007	1.20E-09
rs2736155	6	31,605,199	C	0.044	0.007	3.88E-10
rs10156602	9	96,345,328	A	0.042	0.007	4.36E-09
rs7106434	11	112,860,579	T	0.038	0.007	3.19E-08
rs5803650	13	53,939,598	CT	-0.046	0.008	2.97E-08
rs9513519	13	99,610,146	A	0.039	0.007	3.09E-08

*Abbreviations: Chr* Chromosome, *IBS* Irritable bowel syndrome, *SE* Standard error, *SNP* Single nucleotide polymorphism

### Statistical analysis

We used several methods to estimate the potential causal relationships between gut microbiota and IBS, including fixed/random-effects inverse-variance weighted (IVW) method, weighted median method, MR-Egger regression and MR pleiotropy residual sum and outlier (MR-PRESSO) test. We used the IVW method as the main analysis because it provides the most precise effect estimates and almost all MR-analysis used it as the main analysis [24–26]. The IVW method first calculated the ratio estimates for individual SNPs by using the Wald estimator and Delta method, and then combined the estimates which have been calculated from each SNP, thus obtaining the primary causal estimate [27]. Cochran’s Q test was used to test the heterogeneity among the SNPs we selected, and the random-effects IVW method was chosen if heterogeneity exists (*p* < 0.05) or else fixed-effects IVW method was used [28]. Since the result of IVW method is susceptible to the influences of valid instruments and potential pleiotropic effects, we performed sensitivity analyses to assess the robustness of the association. First, we used the weighted median method to estimate associations since it could provide more reliable estimates of a causal effect when lacking valid instruments [29]. It could provide valid causal effect estimates when less than 50% of information comes from invalid instruments [29]. Second, MR-Egger regression was used to test the potential horizontal pleiotropy, and if the *p*-value of the intercept was less than 0.05, horizontal pleiotropy of SNPs might exist [30]. Finally, we performed the MR-PRESSO test which conducted a global test of heterogeneity to identify if the SNPs existed possible outliers and obtain a corrected association result after removing the potential outliers [31].

To further assess the influence of potential directional pleiotropy, we scanned each of the SNPs used as IVs for their potential secondary phenotypes using the GWAS Catalog (http://www.ebi.ac.uk/gwas, last accessed on November 22, 2022) and performed MR analyses again after excluding the SNPs associated with other phenotypes.

The associations between human gut microbiota and the risk of IBS were presented as odds ratios (ORs) with their 95% confidence intervals (CIs). We corrected for multiple comparisons using the Bonferroni approach at different taxonomic rank and set statistical significance at a different *p*-value (*p*-value < 5.6 × 10^–3^ for Phylum, *p*-value < 3.1 × 10^–3^ for Class, *p*-value < 2.5 × 10^–3^ for Order, *p*-value < 1.6 × 10^–3^ for Family and *p*-value < 4.2 × 10^–4^ for Genus) based on the number of bacterial traits in the specific gut microbiota rank. If a *p*-value was between the significance threshold and 0.05, we considered suggestive evidence for a potential causal association [25]. Only if all MR methods support the association between the gut microbiota and IBS, the reverse MR analysis was performed. All MR analyses were performed using R version 3.6.3 (https://www.r-project.org/) with “Mendelian Randomization”, “TwoSampleMR” and “MR-PRESSO” packages.

## Results

### Main results of the 196 bacterial traits with the risk of IBS

**Fig. 2:**
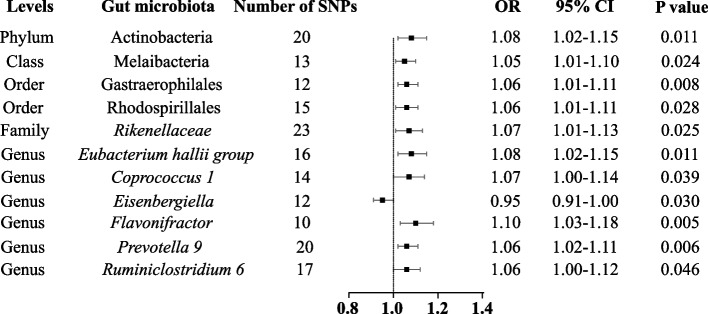
Forest plot of the associations between genetically determined 11 bacterial traits with the risk of irritable bowel syndrome. Abbreviations: CI, confidence interval; OR, odds ratio; SNP, single nucleotide polymorphism

The F-statistics for the 196 bacterial traits were ranged from 21.63 to 144.84, which were all above 10, suggesting less possibility to suffer from weak instrument bias. As for the variances of these 196 bacterial traits explained by the IVs, it was estimated to be ranged from 0.57% to 10.11%. The MR results of the associations between all 196 bacterial traits and the risk of IBS are presented in Additional file 1: Table S1. Briefly, we observed suggestive evidence for 11 bacterial traits to be associated with the risk of IBS using IVW method (Fig. 2). The information of IVs used for these 11 bacterial traits are listed in Additional file 1: Table S2.

**Fig. 3:**
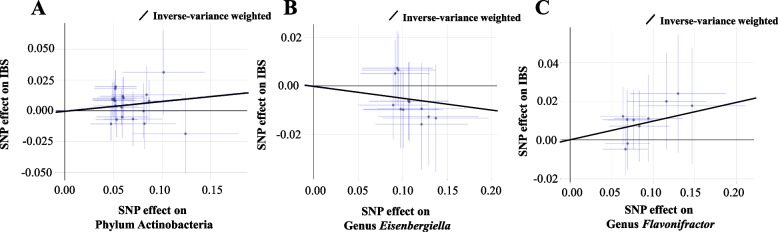
Scatter plot of the associations of genetic variants with three bacterial traits and the risk of irritable bowel syndrome. Abbreviations: IBS, irritable bowel syndrome; MR, mendelian randomization; SNP, single nucleotide polymorphism

In particular, we found that genetically predicted phylum Actinobacteria were positively correlated with the risk of IBS [odds ratio (OR): 1.08; 95% confidence interval (CI): 1.02, 1.15; *p* = 0.011] in the IVW method (Fig. 3). The association between phylum Actinobacteria and IBS remained stable in the weighted-median method (OR: 1.10; 95% CI: 1.01, 1.21; *p* = 0.030). Furthermore, the MR-PRESSO test did not detect any outliers and the results were similar with the primary method (OR: 1.08; 95% CI: 1.00, 1.17; *p* = 0.049). In the MR-Egger regression, there was no evidence of directional pleiotropic effects (intercept *p*-value = 0.270).

As for genus *Flavonifractor*, it was also positively associated with the risk of IBS in IVW method (OR: 1.10; 95% CI: 1.03, 1.18; *p* = 0.005) (Fig. 3). The results from the weighted-median method were consistent (OR: 1.13; 95% CI: 1.03, 1.24; *p* = 0.001). The finding of MR-PRESSO test also supported this result (OR: 1.10; 95% CI: 1.04, 1.16; *p* = 0.008). Intercept of MR-Egger regression also showed no potential horizontal pleiotropy (intercept *p*-value = 0.252).

In contrast, genus *Eisenbergiella* was negatively associated with IBS risk using IVW method (OR: 0.95; 95% CI: 0.91, 1.00; *p* = 0.030) (Fig. 3). In sensitivity analyses, the weighted median method produced similar estimates (OR: 0.92; 95% CI = 0.87, 0.98; *p* = 0.007), though with wider confidence intervals. Additionally, little evidence of directional pleiotropy was found in MR-Egger regression (intercept *p*-value = 0.071) and no outliers were detected with the MR-PRESSO test and the effect estimate was similar (OR: 0.95; 95% CI: 0.91, 0.99; *p* = 0.037).

In addition, we noticed that the rest of eight bacterial traits were suggestively associated with a higher risk of IBS in IVW method (OR: 1.05; 95% CI: 1.01, 1.10; *p* = 0.023 for class Melaibacteria; OR: 1.06; 95% CI: 1.02, 1.11, *p* = 0.008 for order Gastraerophilales; OR: 1.06; 95% CI: 1.01, 1.11; *p* = 0.028 for order Rhodospirillales; OR: 1.07; 95% CI: 1.01, 1.13; *p* = 0.025 for family *Rikenellaceae*; OR: 1.08; 95% CI: 1.02, 1.15; *p* = 0.011 for genus *Eubacterium hallii* group; OR: 1.07; 95% CI: 1.00, 1.14; *p* = 0.039 for genus *Coprococcus* 1; OR: 1.06; 95% CI: 1.02, 1.11, *p* = 0.006 for genus *Prevotella* 9; OR: 1.06; 95% CI: 1.00, 1.12; *p* = 0.046 for genus *Ruminiclostridium* 6), but results from the weighted median method did not support such a causal effect.

To further assess the influence of potential directional pleiotropy on the causal effect estimates, we used the GWAS Catalog to scan the SNPs associated with these 11 bacterial traits and only four SNPs were found to be accompanied with other traits (Table 3). After excluding these pleiotropic SNPs, we recalculated the F-statistics for the updated IV sets, and the associations of phylum Actinobacteria, genus *Eubacterium hallii* group and *Flavonifractor* with the risk of IBS remained stable in the IVW method (OR: 1.08; 95% CI: 1.01, 1.15; *p* = 0.017 for phylum Actinobacteria, F-statistics = 24.18; OR: 1.07; 95% CI: 1.01, 1.14; *p* = 0.021 for genus *Eubacterium hallii* group, F-statistics = 34.11; OR: 1.10; 95% CI: 1.03, 1.19; *p* = 0.007 for genus *Flavonifractor*, F-statistics = 41.76). However, the relationship between genus *Ruminiclostridium* 6 and IBS was unstable (OR: 1.05; 95% CI: 0.99, 1.11; *p* = 0.081, F-statistics = 37.72).

**Table 3:** Details of the genetic variants with potential pleiotropy among instrumental variables used for gut microbiota

Gut microbiota	SNP	Pleiotropic Trait	*p*-value	PMID
Phylum Actinobacteria	rs7570971	Low density lipoprotein cholesterol measurement, alcohol drinking	1.00E-13	30698716
		Total cholesterol measurement	1.00E-13	24097068
		Blood metabolite measurement	8.00E-45	24816252
		Body mass index	5.00E-09	26426971
Genus *Eubacterium hallii* group	rs281379	Pubertal anthropometrics	5.00E-08	23449627
		Crohn's disease	7.00E-12	21102463
		Childhood asthma with severe exacerbations	3.00E-09	33328473
		Alcohol consumption (drinks per week) (MTAG)	4.00E-21	30643251
		Serum levels of protein FUT3	3.00E-16	35078996
Genus *Flavonifractor*	rs6761463	Adult body size	1.00E-11	32376654
Genus *Ruminiclostridium6*	rs11992182	lymphocyte count	2.00E-12	32888494

*From the GWAS Catalog (last assessed on March 22, 2022)

### The result of reverse MR analysis

Finally, we evaluated the potential reverse associations of three bacterial traits and IBS using the reverse MR analyses. We did not find statistically significant associations between IBS and any of these three bacterial traits using IVW method (OR: 1.04; 95% CI: 0.83, 1.31; *p* = 0.692 for phylum Actinobacteria; OR: 0.80; 95% CI: 0.53, 1.21; *p* = 0.290 for genus *Eisenbergiella* and OR: 1.00; 95% CI: 0.74, 1.34; *p* = 0.980 for genus *Flavonifractor*). The results were stable across sensitivity analyses, which are listed in Table 4.

**Table 4:** Effect estimates of the associations of IBS with phylum Actinobacteria, genus *Eisenbergiella* and genus *Flavonifractor* in the reverse MR analyses

Gut microbiome	Methods	N.SNPs	OR	95% CI	*p-*value	Intercept *p- value*
Phylum Actinobacteria						
	Inverse-variance weighted	5	1.05	0.83–1.32	0.693	
	Weighted median	5	1.02	0.77–1.34	0.914	
	MR-PRESSO test	5	1.05	0.91–1.21	0.553	
	MR-Egger	5	/	/	/	0.985
Genus *Eisenbergiella*						
	Inverse-variance weighted	5	0.80	0.53–1.21	0.290	
	Weighted median	5	0.84	0.49–1.44	0.528	
	MR-PRESSO test	5	0.80	0.53–1.21	0.288	
	MR-Egger	5	/	/	/	0.191
Genus *Flavonifractor*						
	Inverse-variance weighted	5	1.00	0.71–1.41	0.982	
	Weighted median	5	1.16	0.79–1.72	0.450	
	MR-PRESSO test	5	1.00	0.70–1.41	0.983	
	MR-Egger	5	/	/	/	0.030

*Abbreviations: CI* Confidence interval, *IBS* Irritable bowel syndrome, *MR* Mendelian randomization, *MR-PRESSO test* MR Pleiotropy RESidual Sum and Outlier test, *OR* Odds ratio, *SNP* Single nucleotide polymorphism

## Discussion

This two-sample MR study identified a total of 11 bacterial taxa, including phylum Actinobacteria, class Melaibacteria, order Gastraerophilales and Rhodospirillales, family *Rikenellaceae*, and genus *Eubacterium hallii* group, *Eisenbergiella*, *Flavonifractor*, *Coprococcus* 1, *Prevotella* 9 and *Ruminiclostridium* 6, might be associated with the risk of IBS. However, sensitivity analyses using different MR methods and restricted IV sets demonstrated three bacterial taxa, *Actinobacteria*, *Flavonifractor*, and *Eisenbergiella*, were associated with the risk of IBS.

Phylum Actinobacteria, one of the major phyla of gut microbiota, is pivotal in the maintenance of gut homeostasis [32]. Disorder of Actinobacteria was associated with several diseases, including inflammatory bowel disease [33], ankylosing spondylitis [34], and type 2 diabetes [35]. A decrease of Actinobacteria was found in patients with IBS compared to healthy controls [36]. The reason might be that Actinobacteria as the initial factor of IBS, the host could produce specific antibodies to reduce the abundance of Actinobacteria after IBS occurring. In addition, the abundance of Actinobacteria showed significant alterations after treatment of IBS [37, 38]. The potential causal relationship between Actinobacteria and IBS observed in this study once again suggested the importance of Actinobacteria in the development of IBS.

Genus *Flavonifractor*, a flavonoid degrader, has also been identified as a risk factor of IBS. The flavonoid compound could alleviate intestinal inflammation of IBS via macrophage-intrinsic AhR [39]. Genus *Flavonifractor* and its species *Flavonifractor plautii* were enriched in the stool communities in children with IBS [40]. In addition, *Flavonifractor plautii* was correlated with recurrent abdominal pain and could elicit enhanced IgG responses in postinfectious IBS patients [41]. Enrichment of the genus *Flavonifractor* was described in adults with comorbid IBS diarrhea-predominant and depression [42]. A previous study also suggested that dietary modifications could decrease the abundance of Flavonifractor to reduce abdominal pain or accelerated transit time in IBS [43]. Taken together, these studies suggested that a high level of Genus *Flavonifractor* may be positively associated with the risk of IBS, which is consistent with our findings.

Genus *Eisenbergiella* was the only identified bacterial taxa being negatively associated with the risk of IBS in this study. However, there was no study reporting the alteration of genus *Eisenbergiella* in IBS patients to date. In animal studies, only one literature reported that genus *Eisenbergiella* showed an increasing trend in the IBS group compared to the control group [44]. Even so, genus *Eisenbergiella* was probably related to eubiosis because it could produce butyrate, acetate, lactate, and succinate as major metabolic products, with a trophic effect on the mucosa [45]. Besides, genus *Eisenbergiella* might be closely related to the reduction in intestinal inflammation in ulcerative colitis mice [46]. Although this study firstly showed a potential causal relationship between genus *Eisenbergiella* and the risk of IBS, further research is needed to explore the underlying biological mechanism between them.

Many previous studies showed that patients with IBS were usually accompanied by gut microbiota dysbiosis, but they were observational studies [9, 47]. This study strengthened the causal effects of gut microbiota on IBS by using a genetic epidemiological approach. In addition, the F-statistic of IVs we used all satisfied the threshold of > 10 which suggested that our analyses were less likely to suffer from weak instrument bias. We further performed a reverse MR analysis that excluded reverse causality. Causal association research will be the future direction of studying the role of gut microbiota in the development of diseases. Nowadays, there were many kinds of research focusing on the role of certain gut bacteria in the disease development using animal models [48, 49]. Our MR analysis results may provide a guide for selecting individual gut bacteria to study the role of gut microbiota in the pathogenesis of IBS.

Nevertheless, our study had several limitations. First, bacterial taxa were only analyzed at the genus level but not at a more specialized level such as species or strain levels. Second, while the majority of the participants enrolled in this GWAS are of European descent, the inclusion of participants with other ethnicities may influence the results. Consequently, the generalization of our findings to other racial groups may be subject to limitations. Third, we selected the IVs for gut microbiota at *p* < 1.0 × 10^−5^ which were larger than traditional genome-wide significance level (*p* < 5 × 10^–8^) to obtain sufficient IVs. In addition, the effect of the bacterial traits we reported was relatively weak and there was no other independent GWAS of IBS with sufficient sample size to validate our findings. Finally, since information of IBS subtypes were not available, further studies are warranted when this information become available.

## Conclusions

In conclusion, this study assessed the potential causal role of gut microbiota on the risk of IBS, and found three bacterial taxa, phylum Actinobacteria, genus *Flavonifractor* and *Eisenbergiella* may have a suggestive causal relationship with the risk of IBS, which may provide clues for the pathogenesis and novel treatment of IBS.

## Supplementary Information

**Additional file 1: Table S1.** Effect estimates of the associations between 196 bacterial traits and the risk of IBS in MR analyses among European populations. **Table S2.** Details of the number of genetic instruments and F-statistic for each cytokine and growth factor.

## Data Availability

The datasets presented in this study can be found in online repositories. The names of the repository/repositories and accession number (PRJNA673102, PRJNA683912, PRJEB14839, EGAS00001004420, PRJEB14839, ERP117287, R000635, EGAS00001001704, EGAS0000100924, SRP097785, ERP016332, PRJEB11532, EGAS00001004869, and ERP015317) can be found in the article [21] or Supplementary Material.

## Abbreviations

CI: Confidence interval
GWAS: Genome-wide association study
IBS: Irritable bowel syndrome
ICD: International classification of diseases
IV: Instrumental variable
IVW: Inverse-variance weighted
LD: Linkage disequilibrium
MR: Mendelian randomization
MR-PRESSO: MR pleiotropy residual sum and outlier
OR: Odds ratio
SNP: Single nucleotide polymorphisms
