# Supplementary material for: Assessing the relationship between gut microbiota and irritable bowel syndrome: a two-sample Mendelian randomization analysis
Source: BMC Gastroenterol. 2023 May 12;23:150. doi: 10.1186/s12876-023-02791-7 (PMC10182631; doi:10.1186/s12876-023-02791-7)
Supplement: Supplementary file 1 — Additional file 1: Table S1. Effect estimates of the associations between 196 bacterial traits and the risk of IBS in MR analyses among European populations. Table S2. Details of the number of genetic instruments and F-statistic for each cytokine and growth factor. [file 12876_2023_2791_MOESM1_ESM.docx]

**Online Supplementary Material**

Bin Liu, et al., Assessing the Relationship Between Gut Microbiota and Irritable bowel syndrome among European Populations: a Two-Sample Mendelian Randomization Analysis

**Table S1** Effect estimates of the associations between 196 bacterial traits and the risk of IBS in MR analyses among European populations

Abbreviations: CI, confidence interval; IBS, irritable bowel syndrome; MR, Mendelian randomization; MR-PRESSO test, MR Pleiotropy RESidual Sum and Outlier test; OR, odds ratio; SNP, single nucleotide polymorphism. **P*-value of the intercept from MR-Egger regression.

**Table S2** Characteristics of the genetic variants associated with 11 bacterial that have been identified to be associated with the risk of IBS among European populations

Abbreviations: Chr, chromosome; IBS, irritable bowel syndrome; SE, standard error; SNP, single nucleotide polymorphism.

**Supplementary Table 1** Effect estimates of the associations between 196 bacterial traits and the risk of IBS in MR analyses^1^

| Gut microbiota | R^2^ | *F* statistic | Methods | N.SNP | OR | 95% CI | *P-*value | Qrs/*P*-interrupt |
| --- | --- | --- | --- | --- | --- | --- | --- | --- |
| Phylum |  |  |  |  |  |  |  |  |
| Actinobacteria |  |  |  |  |  |  |  |  |
|  | 2.80% | 26.43 | Inverse-variance weighted (fixed) | 20 | 1.08 | 1.02-1.15 | 0.011 | 0.077 |
|  |  |  | Weighted median | 20 | 1.10 | 1.01-1.21 | 0.030 |  |
|  |  |  | MR-PRESSO test | 20 | 1.08 | 1.00-1.16 | 0.049 |  |
|  |  |  | MR-Egger | 20 | 0.91 | 0.66-1.25 | 0.558 | 0.270* |
| Bacteroidetes |  |  |  |  |  |  |  |  |
|  | 2.21% | 29.61 | Inverse-variance weighted (fixed) | 14 | 1.04 | 0.97-1.11 | 0.310 | 0.891 |
|  |  |  | Weighted median | 14 | 1.05 | 0.96-1.14 | 0.335 |  |
|  |  |  | MR-PRESSO test | 14 | 1.04 | 0.98-1.09 | 0.196 |  |
|  |  |  | MR-Egger | 14 | 1.02 | 0.88-1.19 | 0.783 | 0.844* |
| Cyanobacteria |  |  |  |  |  |  |  |  |
|  | 3.53% | 67.16 | Inverse-variance weighted (fixed) | 10 | 1.02 | 0.97-1.08 | 0.440 | 0.332 |
|  |  |  | Weighted median | 10 | 0.99 | 0.92-1.07 | 0.796 |  |
|  |  |  | MR-PRESSO test | 10 | 1.02 | 0.97-1.08 | 0.487 |  |
|  |  |  | MR-Egger | 10 | 0.96 | 0.80-1.16 | 0.695 | 0.519* |
| Euryarchaeota |  |  |  |  |  |  |  |  |
|  | 7.39% | 112.50 | Inverse-variance weighted (fixed) | 13 | 1.00 | 0.97-1.04 | 0.818 | 0.497 |
|  |  |  | Weighted median | 13 | 0.98 | 0.93-1.03 | 0.387 |  |
|  |  |  | MR-PRESSO test | 13 | 1.00 | 0.97-1.04 | 0.817 |  |
|  |  |  | MR-Egger | 13 | 1.03 | 0.88-1.20 | 0.736 | 0.770* |
| Firmicutes |  |  |  |  |  |  |  |  |
|  | 2.61% | 24.51 | Inverse-variance weighted (fixed) | 20 | 0.98 | 0.93-1.05 | 0.604 | 0.967 |
|  |  |  | Weighted median | 20 | 0.98 | 0.90-1.06 | 0.554 |  |
|  |  |  | MR-PRESSO test | 20 | 0.98 | 0.94-1.03 | 0.468 |  |
|  |  |  | MR-Egger | 20 | 0.98 | 0.82-1.17 | 0.812 | 0.949* |
| Lentisphaerae |  |  |  |  |  |  |  |  |
|  | 5.25% | 92.39 | Inverse-variance weighted (fixed) | 11 | 0.99 | 0.95-1.03 | 0.562 | 0.920 |
|  |  |  | Weighted median | 11 | 0.97 | 0.92-1.03 | 0.289 |  |
|  |  |  | MR-PRESSO test | 11 | 0.99 | 0.96-1.02 | 0.409 |  |
|  |  |  | MR-Egger | 11 | 1.00 | 0.85-1.18 | 0.984 | 0.857* |
| Proteobacteria |  |  |  |  |  |  |  |  |
|  | 1.77% | 23.61 | Inverse-variance weighted (random) | 14 | 0.99 | 0.92-1.07 | 0.861 | 0.061 |
|  |  |  | Weighted median | 14 | 1.03 | 0.92-1.15 | 0.605 |  |
|  |  |  | MR-PRESSO test | 14 | 0.99 | 0.90-1.09 | 0.894 |  |
|  |  |  | MR-Egger | 14 | 1.02 | 0.75-1.40 | 0.881 | 0.840* |
| Tenericutes |  |  |  |  |  |  |  |  |
|  | 2.60% | 40.77 | Inverse-variance weighted (random) | 12 | 0.95 | 0.87-1.04 | 0.299 | 0.018 |
|  |  |  | Weighted median | 12 | 0.99 | 0.90-1.08 | 0.764 |  |
|  |  |  | MR-PRESSO test | 11 | 0.95 | 0.87-1.04 | 0.321 |  |
|  |  |  | Outlier corrected (MR-PRESSO) | 11 | 0.99 | 0.93-1.05 | 0.788 |  |
|  |  |  | MR-Egger | 12 | 1.04 | 0.76-1.41 | 0.818 | 0.578* |
| Verrucomicrobia |  |  |  |  |  |  |  |  |
|  | 2.42% | 37.94 | Inverse-variance weighted (random) | 12 | 0.99 | 0.93-1.06 | 0.861 | 0.501 |
|  |  |  | Weighted median | 12 | 0.99 | 0.90-1.08 | 0.766 |  |
|  |  |  | MR-PRESSO test | 12 | 0.99 | 0.94-1.06 | 0.860 |  |
|  |  |  | MR-Egger | 12 | 0.96 | 0.81-1.14 | 0.613 | 0.635* |
| Class |  |  |  |  |  |  |  |  |
| Actinobacteria |  |  |  |  |  |  |  |  |
|  | 4.64% | 38.76 | Inverse-variance weighted (fixed) | 23 | 1.02 | 0.97-1.07 | 0.427 | 0.603 |
|  |  |  | Weighted median | 23 | 1.01 | 0.94-1.09 | 0.837 |  |
|  |  |  | MR-PRESSO test | 23 | 1.02 | 0.97-1.07 | 0.410 |  |
|  |  |  | MR-Egger | 23 | 1.05 | 0.91-1.20 | 0.512 | 0.703* |
| Alphaproteobacteria |  |  |  |  |  |  |  |  |
|  | 2.30% | 43.24 | Inverse-variance weighted (fixed) | 10 | 1.02 | 0.96-1.09 | 0.513 | 0.880 |
|  |  |  | Weighted median | 10 | 1.02 | 0.94-1.12 | 0.590 |  |
|  |  |  | MR-PRESSO test | 10 | 1.02 | 0.98-1.07 | 0.376 |  |
|  |  |  | MR-Egger | 10 | 1.07 | 0.83-1.40 | 0.595 | 0.703* |
| Bacilli |  |  |  |  |  |  |  |  |
|  | 3.50% | 30.17 | Inverse-variance weighted (fixed) | 22 | 1.01 | 0.95-1.06 | 0.789 | 0.671 |
|  |  |  | Weighted median | 22 | 0.99 | 0.91-1.06 | 0.710 |  |
|  |  |  | MR-PRESSO test | 22 | 1.01 | 0.96-1.06 | 0.773 |  |
|  |  |  | MR-Egger | 22 | 0.99 | 0.85-1.14 | 0.846 | 0.749* |
| Bacteroidia |  |  |  |  |  |  |  |  |
|  | 2.39% | 28.07 | Inverse-variance weighted (fixed) | 16 | 1.00 | 0.93-1.06 | 0.898 | 0.543 |
|  |  |  | Weighted median | 16 | 0.97 | 0.89-1.07 | 0.589 |  |
|  |  |  | MR-PRESSO test | 16 | 1.00 | 0.94-1.06 | 0.895 |  |
|  |  |  | MR-Egger | 16 | 1.01 | 0.87-1.18 | 0.850 | 0.785* |
|  |  |  |  |  |  |  |  |  |
| Betaproteobacteria | 2.02% | 25.21 | Inverse-variance weighted (fixed) | 15 | 1.02 | 0.95-1.09 | 0.559 | 0.533 |
|  |  |  | Weighted median | 15 | 1.01 | 0.91-1.11 | 0.913 |  |
|  |  |  | MR-PRESSO test | 15 | 1.02 | 0.96-1.09 | 0.552 |  |
|  |  |  | MR-Egger | 15 | 0.97 | 0.77-1.22 | 0.817 | 0.671* |
| Clostridia |  |  |  |  |  |  |  |  |
|  | 1.97% | 21.63 | Inverse-variance weighted (fixed) | 17 | 0.94 | 0.87-1.01 | 0.069 | 0.914 |
|  |  |  | Weighted median | 17 | 0.92 | 0.83-1.01 | 0.066 |  |
|  |  |  | MR-PRESSO test | 17 | 0.94 | 0.89-0.99 | 0.027 |  |
|  |  |  | MR-Egger | 17 | 1.02 | 0.74-1.40 | 0.924 | 0.615* |
| Coriobacteriia |  |  |  |  |  |  |  |  |
|  | 2.99% | 26.93 | Inverse-variance weighted (fixed) | 21 | 1.00 | 0.93-1.08 | 0.955 | 0.018 |
|  |  |  | Weighted median | 21 | 1.02 | 0.94-1.11 | 0.656 |  |
|  |  |  | MR-PRESSO test | 20 | 1.00 | 0.93-1.08 | 0.955 |  |
|  |  |  | MR-Egger | 21 | 0.98 | 0.82-1.18 | 0.849 | 0.814* |
| Deltaproteobacteria |  |  |  |  |  |  |  |  |
|  | 2.22% | 29.78 | Inverse-variance weighted (random) | 14 | 1.00 | 0.92-1.10 | 0.971 | 0.026 |
|  |  |  | Weighted median | 14 | 1.03 | 0.94-1.13 | 0.505 |  |
|  |  |  | MR-PRESSO test | 13 | 1.00 | 0.92-1.10 | 0.971 |  |
|  |  |  | Outlier corrected (MR-PRESSO) | 13 | 0.98 | 0.91-1.05 | 0.565 | 0.234 |
|  |  |  | MR-Egger | 14 | 0.91 | 0.70-1.18 | 0.465 | 0.428* |
| Erysipelotrichia |  |  |  |  |  |  |  |  |
|  | 1.65% | 23.71 | Inverse-variance weighted (fixed) | 13 | 1.06 | 0.98-1.14 | 0.153 | 0.133 |
|  |  |  | Weighted median | 13 | 1.10 | 0.98-1.23 | 0.093 |  |
|  |  |  | MR-PRESSO test | 13 | 1.06 | 0.96-1.16 | 0.259 |  |
|  |  |  | MR-Egger | 13 | 0.91 | 0.61-1.37 | 0.659 | 0.466* |
| Gammaproteobacteria |  |  |  |  |  |  |  |  |
|  | 1.39% | 28.61 | Inverse-variance weighted (fixed) | 9 | 0.96 | 0.88-1.04 | 0.330 | 0.217 |
|  |  |  | Weighted median | 9 | 0.99 | 0.88-1.11 | 0.851 |  |
|  |  |  | MR-PRESSO test | 9 | 0.96 | 0.87-1.06 | 0.425 |  |
|  |  |  | MR-Egger | 9 | 1.18 | 0.85-1.64 | 0.316 | 0.192* |
| Lentisphaeria |  |  |  |  |  |  |  |  |
|  | 4.81% | 92.58 | Inverse-variance weighted (fixed) | 10 | 0.99 | 0.94-1.03 | 0.554 | 0.874 |
|  |  |  | Weighted median | 10 | 0.97 | 0.91-1.03 | 0.302 |  |
|  |  |  | MR-PRESSO test | 10 | 0.99 | 0.96-1.02 | 0.425 |  |
|  |  |  | MR-Egger | 10 | 1.00 | 0.85-1.17 | 0.990 | 0.874* |
| Melainabacteria |  |  |  |  |  |  |  |  |
|  | 5.25% | 78.13 | Inverse-variance weighted (fixed) | 13 | 1.05 | 1.01-1.10 | 0.024 | 0.132 |
|  |  |  | Weighted median | 13 | 1.06 | 0.99-1.13 | 0.078 |  |
|  |  |  | MR-PRESSO test | 13 | 1.05 | 1.00-1.11 | 0.086 |  |
|  |  |  | MR-Egger | 13 | 1.13 | 0.96-1.32 | 0.137 | 0.354* |
| Methanobacteria |  |  |  |  |  |  |  |  |
|  | 7.23% | 119.05 | Inverse-variance weighted (fixed) | 12 | 1.00 | 0.97-1.04 | 0.890 | 0.461 |
|  |  |  | Weighted median | 12 | 0.98 | 0.94-1.03 | 0.402 |  |
|  |  |  | MR-PRESSO test | 12 | 1.00 | 0.97-1.04 | 0.892 |  |
|  |  |  | MR-Egger | 12 | 1.05 | 0.92-1.21 | 0.472 | 0.479* |
| Mollicutes |  |  |  |  |  |  |  |  |
|  | 2.60% | 40.77 | Inverse-variance weighted (random) | 12 | 0.95 | 0.87-1.04 | 0.299 | 0.018 |
|  |  |  | Weighted median | 12 | 0.99 | 0.90-1.08 | 0.764 |  |
|  |  |  | MR-PRESSO test | 11 | 0.95 | 0.87-1.04 | 0.321 |  |
|  |  |  | Outlier corrected (MR-PRESSO) | 11 | 0.99 | 0.93-1.05 | 0.788 |  |
|  |  |  | MR-Egger | 12 | 1.04 | 0.76-1.41 | 0.818 | 0.578* |
| Negativicutes |  |  |  |  |  |  |  |  |
|  | 1.67% | 23.92 | Inverse-variance weighted (fixed) | 13 | 0.96 | 0.89-1.04 | 0.299 | 0.135 |
|  |  |  | Weighted median | 13 | 0.95 | 0.86-1.06 | 0.376 |  |
|  |  |  | MR-PRESSO test | 13 | 0.96 | 0.88-1.05 | 0.406 |  |
|  |  |  | MR-Egger | 13 | 1.09 | 0.80-1.47 | 0.582 | 0.395* |
| Verrucomicrobiae |  |  |  |  |  |  |  |  |
|  | 2.63% | 38.04 | Inverse-variance weighted (fixed) | 13 | 0.99 | 0.94-1.05 | 0.821 | 0.607 |
|  |  |  | Weighted median | 13 | 0.99 | 0.91-1.07 | 0.812 |  |
|  |  |  | MR-PRESSO test | 13 | 0.99 | 0.94-1.05 | 0.810 |  |
|  |  |  | MR-Egger | 13 | 1.08 | 0.87-1.34 | 0.498 | 0.442* |
| Order |  |  |  |  |  |  |  |  |
| Actinomycetales |  |  |  |  |  |  |  |  |
|  | 1.75% | 65.35 | Inverse-variance weighted (fixed) | 5 | 0.97 | 0.90-1.05 | 0.486 | 0.732 |
|  |  |  | Weighted median | 5 | 0.96 | 0.87-1.05 | 0.351 |  |
|  |  |  | MR-PRESSO test | 5 | 0.97 | 0.92-1.03 | 0.382 |  |
|  |  |  | MR-Egger | 5 | 0.93 | 0.76-1.13 | 0.458 | 0.606* |
| Bacillales |  |  |  |  |  |  |  |  |
|  | 7.14% | 128.04 | Inverse-variance weighted (fixed) | 11 | 0.99 | 0.96-1.03 | 0.726 | 0.807 |
|  |  |  | Weighted median | 11 | 1.01 | 0.96-1.05 | 0.793 |  |
|  |  |  | MR-PRESSO test | 11 | 0.99 | 0.97-1.02 | 0.663 |  |
|  |  |  | MR-Egger | 11 | 0.93 | 0.79-1.11 | 0.430 | 0.465* |
| Bacteroidales |  |  |  |  |  |  |  |  |
|  | 2.39% | 28.07 | Inverse-variance weighted (fixed) | 16 | 1.00 | 0.93-1.06 | 0.898 | 0.543 |
|  |  |  | Weighted median | 16 | 0.97 | 0.89-1.07 | 0.589 |  |
|  |  |  | MR-PRESSO test | 16 | 1.00 | 0.94-1.06 | 0.895 |  |
|  |  |  | MR-Egger | 16 | 1.01 | 0.87-1.18 | 0.850 | 0.785* |
| Bifidobacteriales |  |  |  |  |  |  |  |  |
|  | 4.44% | 34.00 | Inverse-variance weighted (fixed) | 25 | 1.05 | 1.00-1.10 | 0.070 | 0.218 |
|  |  |  | Weighted median | 25 | 1.04 | 0.96-1.12 | 0.312 |  |
|  |  |  | MR-PRESSO test | 25 | 1.05 | 0.99-1.11 | 0.112 |  |
|  |  |  | MR-Egger | 25 | 0.92 | 0.74-1.16 | 0.502 | 0.272* |
| Burkholderiales |  |  |  |  |  |  |  |  |
|  | 1.76% | 25.27 | Inverse-variance weighted (fixed) | 13 | 1.05 | 0.97-1.13 | 0.229 | 0.723 |
|  |  |  | Weighted median | 13 | 1.04 | 0.95-1.14 | 0.408 |  |
|  |  |  | MR-PRESSO test | 13 | 1.05 | 0.98-1.11 | 0.184 |  |
|  |  |  | MR-Egger | 13 | 0.96 | 0.76-1.21 | 0.715 | 0.430* |
| Clostridiales |  |  |  |  |  |  |  |  |
|  | 1.97% | 21.66 | Inverse-variance weighted (fixed) | 17 | 0.96 | 0.89-1.03 | 0.238 | 0.766 |
|  |  |  | Weighted median | 17 | 0.97 | 0.88-1.06 | 0.498 |  |
|  |  |  | MR-PRESSO test | 17 | 0.96 | 0.90-1.02 | 0.186 |  |
|  |  |  | MR-Egger | 17 | 0.99 | 0.72-1.36 | 0.934 | 0.858* |
| Coriobacteriales |  |  |  |  |  |  |  |  |
|  | 2.99% | 26.93 | Inverse-variance weighted (random) | 21 | 1.00 | 0.93-1.08 | 0.955 | 0.018 |
|  |  |  | Weighted median | 21 | 1.02 | 0.94-1.11 | 0.656 |  |
|  |  |  | MR-PRESSO test | 21 | 1.00 | 0.93-1.08 | 0.955 |  |
|  |  |  | Outlier corrected (MR-PRESSO) | 20 | 0.99 | 0.92-1.06 | 0.691 |  |
|  |  |  | MR-Egger | 21 | 0.98 | 0.82-1.18 | 0.849 | 0.814* |
| Desulfovibrionales |  |  |  |  |  |  |  |  |
|  | 2.13% | 30.69 | Inverse-variance weighted (random) | 13 | 1.01 | 0.93-1.11 | 0.785 | 0.040 |
|  |  |  | Weighted median | 13 | 1.04 | 0.94-1.15 | 0.490 |  |
|  |  |  | MR-PRESSO test | 13 | 1.01 | 0.93-1.11 | 0.789 |  |
|  |  |  | MR-Egger | 13 | 0.93 | 0.72-1.19 | 0.550 | 0.454* |
| Enterobacteriales |  |  |  |  |  |  |  |  |
|  | 1.84% | 31.17 | Inverse-variance weighted (random) | 11 | 0.98 | 0.87-1.10 | 0.678 | 0.008 |
|  |  |  | Weighted median | 11 | 1.01 | 0.92-1.12 | 0.779 |  |
|  |  |  | MR-PRESSO test | 11 | 0.98 | 0.87-1.10 | 0.687 |  |
|  |  |  | Outlier corrected (MR-PRESSO) | 10 | 1.03 | 0.96-1.11 | 0.475 |  |
|  |  |  | MR-Egger | 11 | 1.12 | 0.65-1.93 | 0.677 | 0.603* |
| Erysipelotrichales |  |  |  |  |  |  |  |  |
|  | 1.65% | 23.71 | Inverse-variance weighted (fixed) | 13 | 1.06 | 0.98-1.14 | 0.153 | 0.133 |
|  |  |  | Weighted median | 13 | 1.10 | 0.98-1.23 | 0.093 |  |
|  |  |  | MR-PRESSO test | 13 | 1.06 | 0.96-1.16 | 0.259 |  |
|  |  |  | MR-Egger | 13 | 0.91 | 0.61-1.37 | 0.659 | 0.466* |
| Gastranaerophilales |  |  |  |  |  |  |  |  |
|  | 4.90% | 78.66 | Inverse-variance weighted (fixed) | 12 | 1.06 | 1.02-1.11 | 0.008 | 0.149 |
|  |  |  | Weighted median | 12 | 1.06 | 1.00-1.13 | 0.054 |  |
|  |  |  | MR-PRESSO test | 12 | 1.06 | 1.01-1.12 | 0.049 |  |
|  |  |  | MR-Egger | 12 | 1.10 | 0.93-1.30 | 0.246 | 0.630* |
| Lactobacillales |  |  |  |  |  |  |  |  |
|  | 3.12% | 31.02 | Inverse-variance weighted (fixed) | 19 | 0.99 | 0.94-1.05 | 0.847 | 0.775 |
|  |  |  | Weighted median | 19 | 0.98 | 0.91-1.06 | 0.594 |  |
|  |  |  | MR-PRESSO test | 19 | 0.99 | 0.95-1.04 | 0.825 |  |
|  |  |  | MR-Egger | 19 | 0.99 | 0.86-1.15 | 0.925 | 0.985* |
| Methanobacteriales |  |  |  |  |  |  |  |  |
|  | 7.23% | 119.05 | Inverse-variance weighted (fixed) | 12 | 1.00 | 0.97-1.04 | 0.890 | 0.461 |
|  |  |  | Weighted median | 12 | 0.98 | 0.94-1.03 | 0.402 |  |
|  |  |  | MR-PRESSO test | 12 | 1.00 | 0.97-1.04 | 0.892 |  |
|  |  |  | MR-Egger | 12 | 1.05 | 0.92-1.21 | 0.472 | 0.479* |
| MollicutesRF9 |  |  |  |  |  |  |  |  |
|  | 3.80% | 42.57 | Inverse-variance weighted (fixed) | 17 | 1.00 | 0.95-1.05 | 0.886 | 0.570 |
|  |  |  | Weighted median | 17 | 0.98 | 0.92-1.06 | 0.663 |  |
|  |  |  | MR-PRESSO test | 17 | 1.00 | 0.95-1.05 | 0.882 |  |
|  |  |  | MR-Egger | 17 | 0.93 | 0.80-1.09 | 0.368 | 0.367* |
| NB1n |  |  |  |  |  |  |  |  |
|  | 8.30% | 92.15 | Inverse-variance weighted (fixed) | 18 | 1.01 | 0.98-1.05 | 0.449 | 0.685 |
|  |  |  | Weighted median | 18 | 1.00 | 0.96-1.05 | 0.872 |  |
|  |  |  | MR-PRESSO test | 18 | 1.01 | 0.98-1.05 | 0.411 |  |
|  |  |  | MR-Egger | 18 | 0.93 | 0.80-1.07 | 0.314 | 0.224* |
| Pasteurellales |  |  |  |  |  |  |  |  |
|  | 5.52% | 56.29 | Inverse-variance weighted (fixed) | 19 | 0.97 | 0.93-1.01 | 0.196 | 0.726 |
|  |  |  | Weighted median | 19 | 0.97 | 0.91-1.02 | 0.245 |  |
|  |  |  | MR-PRESSO test | 19 | 0.97 | 0.94-1.01 | 0.160 |  |
|  |  |  | MR-Egger | 19 | 0.93 | 0.84-1.03 | 0.169 | 0.352* |
| Rhodospirillales |  |  |  |  |  |  |  |  |
|  | 3.84% | 48.72 | Inverse-variance weighted (fixed) | 15 | 1.06 | 1.01-1.11 | 0.028 | 0.314 |
|  |  |  | Weighted median | 15 | 1.03 | 0.96-1.11 | 0.384 |  |
|  |  |  | MR-PRESSO test | 15 | 1.06 | 1.00-1.11 | 0.054 |  |
|  |  |  | MR-Egger | 15 | 1.12 | 0.87-1.45 | 0.361 | 0.623* |
| Selenomonadales |  |  |  |  |  |  |  |  |
|  | 1.67% | 23.92 | Inverse-variance weighted (fixed) | 13 | 0.96 | 0.89-1.04 | 0.299 | 0.135 |
|  |  |  | Weighted median | 13 | 0.95 | 0.86-1.06 | 0.376 |  |
|  |  |  | MR-PRESSO test | 13 | 0.96 | 0.88-1.05 | 0.406 |  |
|  |  |  | MR-Egger | 13 | 1.09 | 0.80-1.47 | 0.582 | 0.395* |
| Verrucomicrobiales |  |  |  |  |  |  |  |  |
|  | 2.63% | 38.04 | Inverse-variance weighted (fixed) | 13 | 0.99 | 0.94-1.05 | 0.821 | 0.607 |
|  |  |  | Weighted median | 13 | 0.99 | 0.91-1.07 | 0.812 |  |
|  |  |  | MR-PRESSO test | 13 | 0.99 | 0.94-1.05 | 0.810 |  |
|  |  |  | MR-Egger | 13 | 1.08 | 0.87-1.34 | 0.498 | 0.442* |
| Victivallales |  |  |  |  |  |  |  |  |
|  | 4.81% | 92.58 | Inverse-variance weighted (fixed) | 10 | 0.99 | 0.94-1.03 | 0.554 | 0.874 |
|  |  |  | Weighted median | 10 | 0.97 | 0.91-1.03 | 0.302 |  |
|  |  |  | MR-PRESSO test | 10 | 0.99 | 0.96-1.02 | 0.425 |  |
|  |  |  | MR-Egger | 10 | 1.00 | 0.85-1.17 | 0.990 | 0.874* |
| Family |  |  |  |  |  |  |  |  |
| Acidaminococcaceae |  |  |  |  |  |  |  |  |
|  | 1.50% | 35.00 | Inverse-variance weighted (fixed) | 8 | 0.99 | 0.91-1.07 | 0.775 | 1.000 |
|  |  |  | Weighted median | 8 | 0.99 | 0.90-1.08 | 0.796 |  |
|  |  |  | MR-PRESSO test | 8 | 0.99 | 0.97-1.01 | 0.274 |  |
|  |  |  | MR-Egger | 8 | 0.98 | 0.78-1.23 | 0.852 | 0.923* |
| Actinomycetaceae |  |  |  |  |  |  |  |  |
|  | 1.76% | 65.52 | Inverse-variance weighted (fixed) | 5 | 0.97 | 0.90-1.05 | 0.484 | 0.733 |
|  |  |  | Weighted median | 5 | 0.96 | 0.87-1.05 | 0.350 |  |
|  |  |  | MR-PRESSO test | 5 | 0.97 | 0.92-1.03 | 0.380 |  |
|  |  |  | MR-Egger | 5 | 0.93 | 0.76-1.13 | 0.455 | 0.603* |
| Alcaligenaceae |  |  |  |  |  |  |  |  |
|  | 2.77% | 26.11 | Inverse-variance weighted (fixed) | 20 | 1.04 | 0.98-1.10 | 0.234 | 0.252 |
|  |  |  | Weighted median | 20 | 1.03 | 0.94-1.12 | 0.583 |  |
|  |  |  | MR-PRESSO test | 20 | 1.04 | 0.97-1.11 | 0.290 |  |
|  |  |  | MR-Egger | 20 | 1.08 | 0.83-1.40 | 0.584 | 0.773* |
| Bacteroidaceae |  |  |  |  |  |  |  |  |
|  | 1.50% | 23.24 | Inverse-variance weighted (random) | 12 | 0.99 | 0.88-1.10 | 0.792 | 0.038 |
|  |  |  | Weighted median | 12 | 0.96 | 0.86-1.07 | 0.471 |  |
|  |  |  | MR-PRESSO test | 12 | 0.99 | 0.88-1.10 | 0.797 |  |
|  |  |  | Outlier corrected (MR-PRESSO) | 11 | 0.95 | 0.87-1.03 | 0.240 | 0.622 |
|  |  |  | MR-Egger | 12 | 0.54 | 0.34-0.86 | 0.009 | 0.009* |
| BacteroidalesS24.7 |  |  |  |  |  |  |  |  |
|  | 3.27% | 56.28 | Inverse-variance weighted (fixed) | 11 | 0.99 | 0.94-1.05 | 0.728 | 0.328 |
|  |  |  | Weighted median | 11 | 1.00 | 0.93-1.08 | 0.978 |  |
|  |  |  | MR-PRESSO test | 11 | 0.99 | 0.93-1.05 | 0.751 |  |
|  |  |  | MR-Egger | 11 | 1.27 | 0.97-1.66 | 0.081 | 0.064* |
| Bifidobacteriaceae |  |  |  |  |  |  |  |  |
|  | 4.44% | 34.00 | Inverse-variance weighted (fixed) | 25 | 1.05 | 1.00-1.10 | 0.070 | 0.218 |
|  |  |  | Weighted median | 25 | 1.04 | 0.96-1.12 | 0.312 |  |
|  |  |  | MR-PRESSO test | 25 | 1.05 | 0.99-1.11 | 0.112 |  |
|  |  |  | MR-Egger | 25 | 0.92 | 0.74-1.16 | 0.502 | 0.272* |
| Christensenellaceae |  |  |  |  |  |  |  |  |
|  | 2.03% | 31.59 | Inverse-variance weighted (fixed) | 12 | 1.00 | 0.93-1.07 | 0.912 | 0.068 |
|  |  |  | Weighted median | 12 | 0.97 | 0.88-1.08 | 0.584 |  |
|  |  |  | MR-PRESSO test | 12 | 1.00 | 0.91-1.09 | 0.934 |  |
|  |  |  | MR-Egger | 12 | 1.13 | 0.94-1.37 | 0.195 | 0.133* |
| Clostridiaceae1 |  |  |  |  |  |  |  |  |
|  | 1.71% | 29.00 | Inverse-variance weighted (fixed) | 11 | 1.01 | 0.94-1.09 | 0.761 | 0.410 |
|  |  |  | Weighted median | 11 | 1.06 | 0.96-1.17 | 0.227 |  |
|  |  |  | MR-PRESSO test | 11 | 1.01 | 0.94-1.09 | 0.771 |  |
|  |  |  | MR-Egger | 11 | 1.14 | 0.92-1.41 | 0.237 | 0.252* |
| ClostridialesvadinBB60 |  |  |  |  |  |  |  |  |
|  | 3.73% | 41.81 | Inverse-variance weighted (fixed) | 17 | 0.99 | 0.95-1.05 | 0.834 | 0.343 |
|  |  |  | Weighted median | 17 | 0.98 | 0.91-1.05 | 0.503 |  |
|  |  |  | MR-PRESSO test | 17 | 0.99 | 0.94-1.05 | 0.844 |  |
|  |  |  | MR-Egger | 17 | 0.98 | 0.84-1.15 | 0.811 | 0.854* |
| Coriobacteriaceae |  |  |  |  |  |  |  |  |
|  | 2.99% | 26.93 | Inverse-variance weighted (random) | 21 | 1.00 | 0.93-1.08 | 0.955 | 0.018 |
|  |  |  | Weighted median | 21 | 1.02 | 0.94-1.11 | 0.656 |  |
|  |  |  | MR-PRESSO test | 20 | 1.00 | 0.93-1.08 | 0.955 |  |
|  |  |  | MR-Egger | 21 | 0.98 | 0.82-1.18 | 0.849 | 0.814* |
| Defluviitaleaceae |  |  |  |  |  |  |  |  |
|  | 3.59% | 52.53 | Inverse-variance weighted (fixed) | 13 | 1.02 | 0.97-1.08 | 0.375 | 0.710 |
|  |  |  | Weighted median | 13 | 1.01 | 0.95-1.08 | 0.721 |  |
|  |  |  | MR-PRESSO test | 13 | 1.02 | 0.98-1.07 | 0.324 |  |
|  |  |  | MR-Egger | 13 | 1.04 | 0.86-1.24 | 0.701 | 0.891* |
| Desulfovibrionaceae |  |  |  |  |  |  |  |  |
|  | 2.00% | 31.20 | Inverse-variance weighted (random) | 12 | 1.01 | 0.92-1.11 | 0.845 | 0.026 |
|  |  |  | Weighted median | 12 | 1.03 | 0.93-1.14 | 0.546 |  |
|  |  |  | MR-PRESSO test | 11 | 1.01 | 0.92-1.11 | 0.848 |  |
|  |  |  | MR-Egger | 12 | 0.93 | 0.71-1.21 | 0.572 | 0.492* |
| Enterobacteriaceae |  |  |  |  |  |  |  |  |
|  | 1.84% | 31.17 | Inverse-variance weighted (random) | 11 | 0.98 | 0.87-1.10 | 0.678 | 0.008 |
|  |  |  | Weighted median | 11 | 1.01 | 0.92-1.12 | 0.779 |  |
|  |  |  | MR-PRESSO test | 11 | 0.98 | 0.87-1.10 | 0.687 |  |
|  |  |  | Outlier corrected (MR-PRESSO) | 10 | 1.03 | 0.96-1.11 | 0.475 | 0.204 |
|  |  |  | MR-Egger | 11 | 1.12 | 0.65-1.93 | 0.677 | 0.603* |
| Erysipelotrichaceae |  |  |  |  |  |  |  |  |
|  | 1.65% | 23.71 | Inverse-variance weighted (fixed) | 13 | 1.06 | 0.98-1.14 | 0.153 | 0.133 |
|  |  |  | Weighted median | 13 | 1.10 | 0.98-1.23 | 0.093 |  |
|  |  |  | MR-PRESSO test | 13 | 1.06 | 0.96-1.16 | 0.259 |  |
|  |  |  | MR-Egger | 13 | 0.91 | 0.61-1.37 | 0.659 | 0.466* |
| FamilyXI (ID: 1936) |  |  |  |  |  |  |  |  |
|  | 6.97% | 137.38 | Inverse-variance weighted (fixed) | 10 | 0.99 | 0.96-1.03 | 0.622 | 0.098 |
|  |  |  | Weighted median | 10 | 0.99 | 0.94-1.04 | 0.566 |  |
|  |  |  | MR-PRESSO test | 10 | 0.99 | 0.95-1.04 | 0.709 |  |
|  |  |  | MR-Egger | 10 | 1.09 | 0.86-1.39 | 0.477 | 0.423* |
| FamilyXIII (ID: 1957) |  |  |  |  |  |  |  |  |
|  | 2.22% | 29.73 | Inverse-variance weighted (fixed) | 14 | 0.99 | 0.92-1.07 | 0.858 | 0.217 |
|  |  |  | Weighted median | 14 | 1.03 | 0.93-1.15 | 0.525 |  |
|  |  |  | MR-PRESSO test | 14 | 0.99 | 0.92-1.08 | 0.877 |  |
|  |  |  | MR-Egger | 14 | 1.06 | 0.78-1.45 | 0.690 | 0.647* |
| Lachnospiraceae |  |  |  |  |  |  |  |  |
|  | 2.61% | 27.33 | Inverse-variance weighted (fixed) | 18 | 0.94 | 0.89-1.00 | 0.059 | 0.275 |
|  |  |  | Weighted median | 18 | 0.98 | 0.89-1.07 | 0.595 |  |
|  |  |  | MR-PRESSO test | 18 | 0.94 | 0.88-1.01 | 0.099 |  |
|  |  |  | MR-Egger | 18 | 0.91 | 0.77-1.08 | 0.283 | 0.673* |
| Lactobacillaceae |  |  |  |  |  |  |  |  |
|  | 4.43% | 65.42 | Inverse-variance weighted (fixed) | 13 | 0.98 | 0.93-1.02 | 0.296 | 0.260 |
|  |  |  | Weighted median | 13 | 0.98 | 0.92-1.04 | 0.456 |  |
|  |  |  | MR-PRESSO test | 13 | 0.98 | 0.93-1.03 | 0.363 |  |
|  |  |  | MR-Egger | 13 | 1.04 | 0.89-1.21 | 0.620 | 0.388* |
| Methanobacteriaceae |  |  |  |  |  |  |  |  |
|  | 7.23% | 119.05 | Inverse-variance weighted (fixed) | 12 | 1.00 | 0.97-1.04 | 0.890 | 0.461 |
|  |  |  | Weighted median | 12 | 0.98 | 0.94-1.03 | 0.402 |  |
|  |  |  | MR-PRESSO test | 12 | 1.00 | 0.97-1.04 | 0.892 |  |
|  |  |  | MR-Egger | 12 | 1.05 | 0.92-1.21 | 0.472 | 0.479* |
| Oxalobacteraceae |  |  |  |  |  |  |  |  |
|  | 6.93% | 90.93 | Inverse-variance weighted (fixed) | 15 | 0.99 | 0.95-1.03 | 0.572 | 0.975 |
|  |  |  | Weighted median | 15 | 0.98 | 0.94-1.03 | 0.492 |  |
|  |  |  | MR-PRESSO test | 15 | 0.99 | 0.97-1.01 | 0.388 |  |
|  |  |  | MR-Egger | 15 | 0.91 | 0.78-1.06 | 0.213 | 0.253* |
| Pasteurellaceae |  |  |  |  |  |  |  |  |
|  | 5.52% | 56.29 | Inverse-variance weighted (fixed) | 19 | 0.97 | 0.93-1.01 | 0.196 | 0.726 |
|  |  |  | Weighted median | 19 | 0.97 | 0.91-1.02 | 0.245 |  |
|  |  |  | MR-PRESSO test | 19 | 0.97 | 0.94-1.01 | 0.160 |  |
|  |  |  | MR-Egger | 19 | 0.93 | 0.84-1.03 | 0.169 | 0.352* |
| Peptococcaceae |  |  |  |  |  |  |  |  |
|  | 2.80% | 52.72 | Inverse-variance weighted (fixed) | 10 | 0.96 | 0.91-1.02 | 0.205 | 0.174 |
|  |  |  | Weighted median | 10 | 0.95 | 0.86-1.03 | 0.215 |  |
|  |  |  | MR-PRESSO test | 10 | 0.96 | 0.90-1.03 | 0.315 |  |
|  |  |  | MR-Egger | 10 | 0.95 | 0.79-1.15 | 0.609 | 0.907* |
| Peptostreptococcaceae |  |  |  |  |  |  |  |  |
|  | 3.05% | 36.03 | Inverse-variance weighted (fixed) | 16 | 1.01 | 0.96-1.07 | 0.607 | 0.597 |
|  |  |  | Weighted median | 16 | 0.97 | 0.90-1.06 | 0.515 |  |
|  |  |  | MR-PRESSO test | 16 | 1.01 | 0.96-1.07 | 0.589 |  |
|  |  |  | MR-Egger | 16 | 0.92 | 0.81-1.05 | 0.228 | 0.110* |
| Porphyromonadaceae |  |  |  |  |  |  |  |  |
|  | 1.46% | 22.60 | Inverse-variance weighted (fixed) | 12 | 1.03 | 0.95-1.12 | 0.465 | 0.243 |
|  |  |  | Weighted median | 12 | 1.05 | 0.94-1.17 | 0.383 |  |
|  |  |  | MR-PRESSO test | 12 | 1.03 | 0.94-1.13 | 0.528 |  |
|  |  |  | MR-Egger | 12 | 1.38 | 0.98-1.96 | 0.069 | 0.089* |
| Prevotellaceae |  |  |  |  |  |  |  |  |
|  | 3.07% | 32.27 | Inverse-variance weighted (random) | 18 | 1.04 | 0.97-1.13 | 0.271 | 0.023 |
|  |  |  | Weighted median | 18 | 1.00 | 0.93-1.09 | 0.917 |  |
|  |  |  | MR-PRESSO test | 18 | 1.04 | 0.97-1.13 | 0.286 |  |
|  |  |  | MR-Egger | 18 | 0.89 | 0.70-1.14 | 0.371 | 0.197* |
| Rhodospirillaceae |  |  |  |  |  |  |  |  |
|  | 4.37% | 49.25 | Inverse-variance weighted (fixed) | 17 | 1.05 | 1.00-1.10 | 0.053 | 0.566 |
|  |  |  | Weighted median | 17 | 1.04 | 0.97-1.10 | 0.268 |  |
|  |  |  | MR-PRESSO test | 17 | 1.05 | 1.00-1.09 | 0.058 |  |
|  |  |  | MR-Egger | 17 | 1.08 | 0.87-1.35 | 0.485 | 0.766* |
| Rikenellaceae |  |  |  |  |  |  |  |  |
|  | 3.07% | 25.20 | Inverse-variance weighted (fixed) | 23 | 1.07 | 1.01-1.13 | 0.025 | 0.176 |
|  |  |  | Weighted median | 23 | 1.07 | 0.98-1.16 | 0.125 |  |
|  |  |  | MR-PRESSO test | 23 | 1.07 | 1.00-1.14 | 0.055 |  |
|  |  |  | MR-Egger | 23 | 1.15 | 0.93-1.41 | 0.194 | 0.470* |
| Ruminococcaceae |  |  |  |  |  |  |  |  |
|  | 1.90% | 29.58 | Inverse-variance weighted (fixed) | 12 | 1.03 | 0.96-1.10 | 0.473 | 0.591 |
|  |  |  | Weighted median | 12 | 1.04 | 0.94-1.15 | 0.499 |  |
|  |  |  | MR-PRESSO test | 12 | 1.03 | 0.96-1.10 | 0.452 |  |
|  |  |  | MR-Egger | 12 | 0.94 | 0.81-1.11 | 0.479 | 0.244* |
| Streptococcaceae |  |  |  |  |  |  |  |  |
|  | 2.70% | 26.71 | Inverse-variance weighted (fixed) | 19 | 1.02 | 0.96-1.08 | 0.589 | 0.157 |
|  |  |  | Weighted median | 19 | 1.02 | 0.93-1.11 | 0.656 |  |
|  |  |  | MR-PRESSO test | 19 | 1.02 | 0.95-1.09 | 0.645 |  |
|  |  |  | MR-Egger | 19 | 1.01 | 0.78-1.30 | 0.967 | 0.931* |
| Veillonellaceae |  |  |  |  |  |  |  |  |
|  | 3.79% | 34.40 | Inverse-variance weighted (random) | 21 | 1.00 | 0.92-1.08 | 0.924 | 0.000 |
|  |  |  | Weighted median | 21 | 1.05 | 0.97-1.13 | 0.215 |  |
|  |  |  | MR-PRESSO test | 21 | 1.00 | 0.92-1.08 | 0.925 |  |
|  |  |  | Outlier corrected (MR-PRESSO) | 19 | 1.03 | 0.96-1.10 | 0.395 |  |
|  |  |  | MR-Egger | 21 | 1.18 | 1.01-1.38 | 0.041 | 0.020* |
| Verrucomicrobiaceae |  |  |  |  |  |  |  |  |
|  | 2.63% | 38.04 | Inverse-variance weighted (fixed) | 13 | 0.99 | 0.94-1.05 | 0.821 | 0.607 |
|  |  |  | Weighted median | 13 | 0.99 | 0.91-1.07 | 0.811 |  |
|  |  |  | MR-PRESSO test | 13 | 0.99 | 0.94-1.05 | 0.809 |  |
|  |  |  | MR-Egger | 13 | 1.08 | 0.87-1.34 | 0.500 | 0.443* |
| Victivallaceae |  |  |  |  |  |  |  |  |
|  | 9.17% | 123.35 | Inverse-variance weighted (fixed) | 15 | 1.01 | 0.98-1.05 | 0.386 | 0.199 |
|  |  |  | Weighted median | 15 | 0.98 | 0.94-1.03 | 0.395 |  |
|  |  |  | MR-PRESSO test | 15 | 1.01 | 0.98-1.05 | 0.459 |  |
|  |  |  | MR-Egger | 15 | 0.97 | 0.80-1.16 | 0.709 | 0.592* |
| Genus |  |  |  |  |  |  |  |  |
| Clostridiuminnocuum |  |  |  |  |  |  |  |  |
|  | 5.90% | 104.48 | Inverse-variance weighted (fixed) | 11 | 0.99 | 0.95-1.03 | 0.687 | 0.171 |
|  |  |  | Weighted median | 11 | 0.98 | 0.92-1.04 | 0.436 |  |
|  |  |  | MR-PRESSO test | 11 | 0.99 | 0.94-1.04 | 0.741 |  |
|  |  |  | MR-Egger | 11 | 0.79 | 0.62-1 | 0.053 | 0.058* |
| Eubacteriumbrachy |  |  |  |  |  |  |  |  |
|  | 5.96% | 105.62 | Inverse-variance weighted (fixed) | 11 | 1.00 | 0.96-1.04 | 0.981 | 0.602 |
|  |  |  | Weighted median | 11 | 1.00 | 0.95-1.06 | 0.931 |  |
|  |  |  | MR-PRESSO test | 11 | 1.00 | 0.96-1.04 | 0.980 |  |
|  |  |  | MR-Egger | 11 | 1.05 | 0.92-1.21 | 0.466 | 0.443* |
| Eubacteriumcoprostanoligenes |  |  |  |  |  |  |  |  |
|  | 1.95% | 24.32 | Inverse-variance weighted (fixed) | 15 | 0.95 | 0.88-1.02 | 0.135 | 0.193 |
|  |  |  | Weighted median | 15 | 0.94 | 0.85-1.04 | 0.222 |  |
|  |  |  | MR-PRESSO test | 15 | 0.95 | 0.87-1.03 | 0.213 |  |
|  |  |  | MR-Egger | 15 | 1.12 | 0.81-1.54 | 0.506 | 0.305* |
| Eubacteriumeligens |  |  |  |  |  |  |  |  |
|  | 1.85% | 31.49 | Inverse-variance weighted (fixed) | 11 | 0.99 | 0.92-1.06 | 0.768 | 0.078 |
|  |  |  | Weighted median | 11 | 1.04 | 0.94-1.14 | 0.424 |  |
|  |  |  | MR-PRESSO test | 11 | 0.99 | 0.90-1.09 | 0.825 |  |
|  |  |  | MR-Egger | 11 | 1.13 | 0.89-1.44 | 0.328 | 0.250* |
| Eubacteriumfissicatena |  |  |  |  |  |  |  |  |
|  | 5.01% | 107.36 | Inverse-variance weighted (fixed) | 9 | 0.98 | 0.94-1.02 | 0.329 | 0.055 |
|  |  |  | Weighted median | 9 | 0.98 | 0.92-1.05 | 0.538 |  |
|  |  |  | MR-PRESSO test | 9 | 0.98 | 0.92-1.04 | 0.499 |  |
|  |  |  | MR-Egger | 9 | 1.10 | 0.79-1.54 | 0.570 | 0.480* |
| Eubacteriumhallii |  |  |  |  |  |  |  |  |
|  | 2.82% | 33.25 | Inverse-variance weighted (fixed) | 16 | 1.08 | 1.02-1.15 | 0.011 | 0.667 |
|  |  |  | Weighted median | 16 | 1.05 | 0.96-1.14 | 0.314 |  |
|  |  |  | MR-PRESSO test | 16 | 1.08 | 1.02-1.14 | 0.013 |  |
|  |  |  | MR-Egger | 16 | 1.06 | 0.93-1.20 | 0.380 | 0.706* |
| Eubacteriumnodatum |  |  |  |  |  |  |  |  |
|  | 7.15% | 128.36 | Inverse-variance weighted (fixed) | 11 | 1.00 | 0.97-1.04 | 0.929 | 0.320 |
|  |  |  | Weighted median | 11 | 0.99 | 0.95-1.04 | 0.807 |  |
|  |  |  | MR-PRESSO test | 11 | 1.00 | 0.96-1.04 | 0.935 |  |
|  |  |  | MR-Egger | 11 | 0.91 | 0.77-1.07 | 0.249 | 0.229* |
| Eubacteriumoxidoreducens |  |  |  |  |  |  |  |  |
|  | 2.64% | 82.71 | Inverse-variance weighted (random) | 6 | 1.01 | 0.90-1.15 | 0.818 | 0.001 |
|  |  |  | Weighted median | 6 | 1.06 | 0.96-1.17 | 0.235 |  |
|  |  |  | MR-PRESSO test | 4 | 1.01 | 0.90-1.15 | 0.827 |  |
|  |  |  | Outlier corrected (MR-PRESSO) | 4 | 1.01 | 0.94-1.09 | 0.760 |  |
|  |  |  | MR-Egger | 6 | 1.38 | 0.93-2.03 | 0.109 | 0.111* |
| Eubacteriumrectale |  |  |  |  |  |  |  |  |
|  | 1.78% | 25.52 | Inverse-variance weighted (fixed) | 13 | 1.05 | 0.97-1.13 | 0.208 | 0.122 |
|  |  |  | Weighted median | 13 | 1.05 | 0.95-1.17 | 0.330 |  |
|  |  |  | MR-PRESSO test | 13 | 1.05 | 0.96-1.14 | 0.322 |  |
|  |  |  | MR-Egger | 13 | 1.19 | 0.92-1.54 | 0.178 | 0.291* |
| Eubacteriumruminantium |  |  |  |  |  |  |  |  |
|  | 5.88% | 60.26 | Inverse-variance weighted (fixed) | 19 | 1.02 | 0.98-1.07 | 0.264 | 0.343 |
|  |  |  | Weighted median | 19 | 1.03 | 0.97-1.09 | 0.381 |  |
|  |  |  | MR-PRESSO test | 19 | 1.02 | 0.98-1.07 | 0.301 |  |
|  |  |  | MR-Egger | 19 | 0.98 | 0.83-1.15 | 0.795 | 0.568* |
| Eubacteriumventriosum |  |  |  |  |  |  |  |  |
|  | 2.60% | 28.78 | Inverse-variance weighted (random) | 17 | 0.97 | 0.89-1.06 | 0.536 | 0.018 |
|  |  |  | Weighted median | 17 | 0.99 | 0.90-1.09 | 0.822 |  |
|  |  |  | MR-PRESSO test | 16 | 0.97 | 0.89-1.06 | 0.545 |  |
|  |  |  | Outlier corrected (MR-PRESSO) | 16 | 0.94 | 0.88-1.01 | 0.129 |  |
|  |  |  | MR-Egger | 17 | 1.05 | 0.70-1.57 | 0.805 | 0.695* |
| Eubacteriumxylanophilum |  |  |  |  |  |  |  |  |
|  | 2.11% | 32.91 | Inverse-variance weighted (fixed) | 12 | 0.96 | 0.90-1.03 | 0.295 | 0.334 |
|  |  |  | Weighted median | 12 | 0.99 | 0.90-1.09 | 0.779 |  |
|  |  |  | MR-PRESSO test | 12 | 0.96 | 0.90-1.04 | 0.345 |  |
|  |  |  | MR-Egger | 12 | 1.09 | 0.86-1.38 | 0.480 | 0.287* |
| Ruminococcusgauvreauii |  |  |  |  |  |  |  |  |
|  | 2.15% | 31.00 | Inverse-variance weighted (fixed) | 13 | 1.06 | 0.99-1.13 | 0.094 | 0.299 |
|  |  |  | Weighted median | 13 | 1.04 | 0.95-1.15 | 0.359 |  |
|  |  |  | MR-PRESSO test | 13 | 1.06 | 0.99-1.14 | 0.147 |  |
|  |  |  | MR-Egger | 13 | 1.13 | 0.80-1.60 | 0.483 | 0.697* |
| Ruminococcusgnavus |  |  |  |  |  |  |  |  |
|  | 4.08% | 64.94 | Inverse-variance weighted (fixed) | 12 | 1.02 | 0.97-1.07 | 0.440 | 0.198 |
|  |  |  | Weighted median | 12 | 1.00 | 0.94-1.07 | 0.968 |  |
|  |  |  | MR-PRESSO test | 12 | 1.02 | 0.96-1.08 | 0.518 |  |
|  |  |  | MR-Egger | 12 | 1.12 | 0.87-1.45 | 0.376 | 0.446* |
| Ruminococcustorques |  |  |  |  |  |  |  |  |
|  | 2.08% | 26.00 | Inverse-variance weighted (fixed) | 15 | 0.99 | 0.93-1.06 | 0.780 | 0.574 |
|  |  |  | Weighted median | 15 | 0.98 | 0.89-1.08 | 0.755 |  |
|  |  |  | MR-PRESSO test | 15 | 0.99 | 0.93-1.05 | 0.771 |  |
|  |  |  | MR-Egger | 15 | 0.96 | 0.79-1.17 | 0.697 | 0.755* |
| Actinomyces |  |  |  |  |  |  |  |  |
|  | 2.66% | 62.61 | Inverse-variance weighted (fixed) | 8 | 1.00 | 0.94-1.07 | 0.918 | 0.694 |
|  |  |  | Weighted median | 8 | 1.01 | 0.93-1.09 | 0.890 |  |
|  |  |  | MR-PRESSO test | 8 | 1.00 | 0.95-1.05 | 0.903 |  |
|  |  |  | MR-Egger | 8 | 0.93 | 0.78-1.11 | 0.440 | 0.388* |
| Adlercreutzia |  |  |  |  |  |  |  |  |
|  | 3.11% | 49.06 | Inverse-variance weighted (fixed) | 12 | 1.01 | 0.95-1.07 | 0.773 | 0.117 |
|  |  |  | Weighted median | 12 | 1.03 | 0.95-1.11 | 0.530 |  |
|  |  |  | MR-PRESSO test | 12 | 1.01 | 0.94-1.08 | 0.819 |  |
|  |  |  | MR-Egger | 12 | 1.22 | 0.88-1.68 | 0.231 | 0.241* |
| Akkermansia |  |  |  |  |  |  |  |  |
|  | 2.63% | 38.06 | Inverse-variance weighted (fixed) | 13 | 0.99 | 0.94-1.05 | 0.820 | 0.607 |
|  |  |  | Weighted median | 13 | 0.99 | 0.91-1.07 | 0.810 |  |
|  |  |  | MR-PRESSO test | 13 | 0.99 | 0.94-1.05 | 0.808 |  |
|  |  |  | MR-Egger | 13 | 1.08 | 0.87-1.34 | 0.503 | 0.446* |
| Alistipes |  |  |  |  |  |  |  |  |
|  | 1.80% | 22.37 | Inverse-variance weighted (fixed) | 15 | 0.97 | 0.91-1.05 | 0.486 | 0.079 |
|  |  |  | Weighted median | 15 | 0.98 | 0.88-1.10 | 0.776 |  |
|  |  |  | MR-PRESSO test | 15 | 0.97 | 0.89-1.07 | 0.587 |  |
|  |  |  | MR-Egger | 15 | 0.91 | 0.56-1.46 | 0.681 | 0.756* |
| Allisonella |  |  |  |  |  |  |  |  |
|  | 6.06% | 131.44 | Inverse-variance weighted (fixed) | 9 | 1.02 | 0.98-1.06 | 0.248 | 0.859 |
|  |  |  | Weighted median | 9 | 1.02 | 0.98-1.07 | 0.343 |  |
|  |  |  | MR-PRESSO test | 9 | 1.02 | 1.00-1.05 | 0.140 |  |
|  |  |  | MR-Egger | 9 | 1.11 | 0.86-1.43 | 0.415 | 0.518* |
| Alloprevotella |  |  |  |  |  |  |  |  |
|  | 4.85% | 133.35 | Inverse-variance weighted (fixed) | 7 | 0.99 | 0.94-1.03 | 0.520 | 0.958 |
|  |  |  | Weighted median | 7 | 1.00 | 0.94-1.06 | 0.960 |  |
|  |  |  | MR-PRESSO test | 7 | 0.99 | 0.96-1.01 | 0.250 |  |
|  |  |  | MR-Egger | 7 | 0.89 | 0.59-1.34 | 0.581 | 0.627* |
| Anaerofilum |  |  |  |  |  |  |  |  |
|  | 5.45% | 88.11 | Inverse-variance weighted (fixed) | 12 | 1.00 | 0.96-1.04 | 0.902 | 0.240 |
|  |  |  | Weighted median | 12 | 0.99 | 0.93-1.05 | 0.689 |  |
|  |  |  | MR-PRESSO test | 12 | 1.00 | 0.95-1.05 | 0.915 |  |
|  |  |  | MR-Egger | 12 | 0.89 | 0.72-1.10 | 0.294 | 0.293* |
| Anaerostipes |  |  |  |  |  |  |  |  |
|  | 2.19% | 27.40 | Inverse-variance weighted (fixed) | 15 | 0.95 | 0.89-1.02 | 0.163 | 0.054 |
|  |  |  | Weighted median | 15 | 0.91 | 0.82-1.01 | 0.070 |  |
|  |  |  | MR-PRESSO test | 15 | 0.95 | 0.87-1.04 | 0.299 |  |
|  |  |  | MR-Egger | 15 | 1.01 | 0.78-1.31 | 0.941 | 0.64* |
| Anaerotruncus |  |  |  |  |  |  |  |  |
|  | 2.14% | 25.10 | Inverse-variance weighted (fixed) | 15 | 0.95 | 0.89-1.02 | 0.174 | 0.374 |
|  |  |  | Weighted median | 15 | 0.92 | 0.84-1.01 | 0.093 |  |
|  |  |  | MR-PRESSO test | 15 | 0.95 | 0.89-1.02 | 0.211 |  |
|  |  |  | MR-Egger | 15 | 0.89 | 0.70-1.12 | 0.319 | 0.529* |
| Bacteroides |  |  |  |  |  |  |  |  |
|  | 1.50% | 23.24 | Inverse-variance weighted (random) | 12 | 0.99 | 0.88-1.10 | 0.792 | 0.038 |
|  |  |  | Weighted median | 12 | 0.96 | 0.86-1.07 | 0.471 |  |
|  |  |  | MR-PRESSO test | 12 | 0.99 | 0.88-1.10 | 0.797 |  |
|  |  |  | Outlier corrected (MR-PRESSO) | 11 | 0.95 | 0.87-1.03 | 0.240 |  |
|  |  |  | MR-Egger | 12 | 0.54 | 0.34-0.86 | 0.009 | 0.009* |
| Barnesiella |  |  |  |  |  |  |  |  |
|  | 2.82% | 31.24 | Inverse-variance weighted (fixed) | 17 | 1.04 | 0.98-1.10 | 0.193 | 0.515 |
|  |  |  | Weighted median | 17 | 1.03 | 0.95-1.12 | 0.442 |  |
|  |  |  | MR-PRESSO test | 17 | 1.04 | 0.98-1.10 | 0.199 |  |
|  |  |  | MR-Egger | 17 | 1.08 | 0.87-1.33 | 0.498 | 0.745* |
| Bifidobacterium |  |  |  |  |  |  |  |  |
|  | 4.32% | 37.58 | Inverse-variance weighted (random) | 22 | 1.06 | 0.99-1.13 | 0.111 | 0.023 |
|  |  |  | Weighted median | 22 | 1.06 | 0.98-1.15 | 0.152 |  |
|  |  |  | MR-PRESSO test | 22 | 1.06 | 0.99-1.13 | 0.126 |  |
|  |  |  | MR-Egger | 22 | 1.09 | 0.88-1.33 | 0.429 | 0.769* |
| Bilophila |  |  |  |  |  |  |  |  |
|  | 2.82% | 31.30 | Inverse-variance weighted (fixed) | 17 | 0.96 | 0.91-1.02 | 0.192 | 0.155 |
|  |  |  | Weighted median | 17 | 0.96 | 0.89-1.04 | 0.362 |  |
|  |  |  | MR-PRESSO test | 17 | 0.96 | 0.90-1.03 | 0.279 |  |
|  |  |  | MR-Egger | 17 | 0.94 | 0.72-1.24 | 0.669 | 0.874* |
| Blautia |  |  |  |  |  |  |  |  |
|  | 2.01% | 28.89 | Inverse-variance weighted (fixed) | 13 | 1.00 | 0.93-1.07 | 0.933 | 0.904 |
|  |  |  | Weighted median | 13 | 0.98 | 0.89-1.08 | 0.729 |  |
|  |  |  | MR-PRESSO test | 13 | 1.00 | 0.95-1.05 | 0.909 |  |
|  |  |  | MR-Egger | 13 | 1.09 | 0.94-1.26 | 0.240 | 0.17* |
| Butyricicoccus |  |  |  |  |  |  |  |  |
|  | 1.95% | 40.56 | Inverse-variance weighted (fixed) | 9 | 0.98 | 0.91-1.05 | 0.519 | 0.319 |
|  |  |  | Weighted median | 9 | 0.97 | 0.88-1.06 | 0.471 |  |
|  |  |  | MR-PRESSO test | 9 | 0.98 | 0.90-1.05 | 0.566 |  |
|  |  |  | MR-Egger | 9 | 0.99 | 0.85-1.15 | 0.888 | 0.851* |
| Butyricimonas |  |  |  |  |  |  |  |  |
|  | 3.92% | 41.48 | Inverse-variance weighted (fixed) | 18 | 0.95 | 0.91-1.00 | 0.052 | 0.348 |
|  |  |  | Weighted median | 18 | 0.95 | 0.89-1.02 | 0.156 |  |
|  |  |  | MR-PRESSO test | 18 | 0.95 | 0.90-1.00 | 0.081 |  |
|  |  |  | MR-Egger | 18 | 0.93 | 0.77-1.14 | 0.495 | 0.849* |
| Butyrivibrio |  |  |  |  |  |  |  |  |
|  | 10.11% | 128.73 | Inverse-variance weighted (fixed) | 16 | 1.01 | 0.98-1.04 | 0.712 | 0.111 |
|  |  |  | Weighted median | 16 | 0.99 | 0.95-1.04 | 0.722 |  |
|  |  |  | MR-PRESSO test | 16 | 1.01 | 0.97-1.04 | 0.764 |  |
|  |  |  | MR-Egger | 16 | 1.01 | 0.85-1.19 | 0.953 | 0.995* |
| CandidatusSoleaferrea |  |  |  |  |  |  |  |  |
|  | 5.83% | 70.84 | Inverse-variance weighted (fixed) | 16 | 1.00 | 0.96-1.04 | 0.997 | 0.266 |
|  |  |  | Weighted median | 16 | 0.97 | 0.92-1.03 | 0.395 |  |
|  |  |  | MR-PRESSO test | 16 | 1.00 | 0.96-1.05 | 0.997 |  |
|  |  |  | MR-Egger | 16 | 0.97 | 0.80-1.19 | 0.786 | 0.78* |
| Catenibacterium |  |  |  |  |  |  |  |  |
|  | 3.28% | 124.43 | Inverse-variance weighted (fixed) | 5 | 0.98 | 0.93-1.04 | 0.549 | 0.999 |
|  |  |  | Weighted median | 5 | 0.98 | 0.92-1.05 | 0.578 |  |
|  |  |  | MR-PRESSO test | 5 | 0.98 | 0.98-0.99 | 0.010 |  |
|  |  |  | MR-Egger | 5 | 0.96 | 0.60-1.55 | 0.878 | 0.929* |
| ChristensenellaceaeR.7 |  |  |  |  |  |  |  |  |
|  | 1.50% | 25.33 | Inverse-variance weighted (fixed) | 11 | 0.97 | 0.89-1.05 | 0.402 | 0.186 |
|  |  |  | Weighted median | 11 | 0.93 | 0.83-1.04 | 0.232 |  |
|  |  |  | MR-PRESSO test | 11 | 0.97 | 0.88-1.06 | 0.491 |  |
|  |  |  | MR-Egger | 11 | 1.12 | 0.84-1.49 | 0.441 | 0.287* |
| Clostridiumsensustricto1 |  |  |  |  |  |  |  |  |
|  | 1.86% | 38.54 | Inverse-variance weighted (fixed) | 9 | 1.02 | 0.95-1.09 | 0.636 | 0.232 |
|  |  |  | Weighted median | 9 | 1.07 | 0.98-1.18 | 0.145 |  |
|  |  |  | MR-PRESSO test | 9 | 1.02 | 0.94-1.10 | 0.690 |  |
|  |  |  | MR-Egger | 9 | 1.13 | 0.95-1.35 | 0.155 | 0.17* |
| Collinsella |  |  |  |  |  |  |  |  |
|  | 1.93% | 27.67 | Inverse-variance weighted (random) | 13 | 0.95 | 0.86-1.05 | 0.300 | 0.013 |
|  |  |  | Weighted median | 13 | 0.94 | 0.85-1.04 | 0.264 |  |
|  |  |  | MR-PRESSO test | 12 | 0.95 | 0.86-1.05 | 0.320 |  |
|  |  |  | Outlier corrected (MR-PRESSO) | 12 | 0.92 | 0.84-1.00 | 0.062 |  |
|  |  |  | MR-Egger | 13 | 0.82 | 0.54-1.27 | 0.377 | 0.510* |
| Coprobacter |  |  |  |  |  |  |  |  |
|  | 5.29% | 73.18 | Inverse-variance weighted (fixed) | 14 | 1.01 | 0.97-1.06 | 0.574 | 0.925 |
|  |  |  | Weighted median | 14 | 1.02 | 0.96-1.08 | 0.545 |  |
|  |  |  | MR-PRESSO test | 14 | 1.01 | 0.98-1.04 | 0.441 |  |
|  |  |  | MR-Egger | 14 | 1.00 | 0.88-1.14 | 0.988 | 0.829* |
| Coprococcus1 |  |  |  |  |  |  |  |  |
|  | 2.38% | 31.91 | Inverse-variance weighted (fixed) | 14 | 1.07 | 1.00-1.14 | 0.039 | 0.117 |
|  |  |  | Weighted median | 14 | 1.09 | 0.99-1.20 | 0.090 |  |
|  |  |  | MR-PRESSO test | 14 | 1.07 | 0.99-1.16 | 0.113 |  |
|  |  |  | MR-Egger | 14 | 1.03 | 0.84-1.26 | 0.782 | 0.678* |
| Coprococcus2 |  |  |  |  |  |  |  |  |
|  | 2.36% | 36.88 | Inverse-variance weighted (fixed) | 12 | 1.03 | 0.96-1.10 | 0.388 | 0.336 |
|  |  |  | Weighted median | 12 | 1.05 | 0.95-1.15 | 0.342 |  |
|  |  |  | MR-PRESSO test | 12 | 1.03 | 0.96-1.10 | 0.433 |  |
|  |  |  | MR-Egger | 12 | 0.90 | 0.63-1.28 | 0.554 | 0.445* |
| Coprococcus3 |  |  |  |  |  |  |  |  |
|  | 1.76% | 27.40 | Inverse-variance weighted (fixed) | 12 | 1.03 | 0.95-1.10 | 0.510 | 0.876 |
|  |  |  | Weighted median | 12 | 1.01 | 0.92-1.11 | 0.866 |  |
|  |  |  | MR-PRESSO test | 12 | 1.03 | 0.97-1.08 | 0.390 |  |
|  |  |  | MR-Egger | 12 | 1.08 | 0.77-1.52 | 0.645 | 0.745* |
| DefluviitaleaceaeUCG011 |  |  |  |  |  |  |  |  |
|  | 3.18% | 54.72 | Inverse-variance weighted (fixed) | 11 | 1.01 | 0.95-1.06 | 0.827 | 0.479 |
|  |  |  | Weighted median | 11 | 1.01 | 0.94-1.09 | 0.763 |  |
|  |  |  | MR-PRESSO test | 11 | 1.01 | 0.95-1.06 | 0.827 |  |
|  |  |  | MR-Egger | 11 | 1.06 | 0.86-1.30 | 0.578 | 0.605* |
| Desulfovibrio |  |  |  |  |  |  |  |  |
|  | 3.18% | 50.13 | Inverse-variance weighted (fixed) | 12 | 0.97 | 0.91-1.02 | 0.243 | 0.727 |
|  |  |  | Weighted median | 12 | 0.97 | 0.90-1.04 | 0.360 |  |
|  |  |  | MR-PRESSO test | 12 | 0.97 | 0.92-1.01 | 0.194 |  |
|  |  |  | MR-Egger | 12 | 0.96 | 0.81-1.15 | 0.696 | 0.987* |
| Dialister |  |  |  |  |  |  |  |  |
|  | 2.16% | 33.65 | Inverse-variance weighted (fixed) | 12 | 0.98 | 0.92-1.05 | 0.627 | 0.108 |
|  |  |  | Weighted median | 12 | 1.00 | 0.92-1.09 | 0.979 |  |
|  |  |  | MR-PRESSO test | 12 | 0.98 | 0.91-1.07 | 0.704 |  |
|  |  |  | MR-Egger | 12 | 0.97 | 0.68-1.39 | 0.874 | 0.942* |
| Dorea |  |  |  |  |  |  |  |  |
|  | 1.82% | 26.17 | Inverse-variance weighted (random) | 13 | 1.02 | 0.92-1.13 | 0.698 | 0.015 |
|  |  |  | Weighted median | 13 | 1.05 | 0.94-1.16 | 0.376 |  |
|  |  |  | MR-PRESSO test | 12 | 1.02 | 0.92-1.13 | 0.704 |  |
|  |  |  | Outlier corrected (MR-PRESSO) | 12 | 0.98 | 0.90-1.07 | 0.658 |  |
|  |  |  | MR-Egger | 13 | 1.11 | 0.83-1.48 | 0.485 | 0.547* |
| Eggerthella |  |  |  |  |  |  |  |  |
|  | 4.33% | 75.46 | Inverse-variance weighted (fixed) | 11 | 0.99 | 0.95-1.04 | 0.743 | 0.977 |
|  |  |  | Weighted median | 11 | 0.98 | 0.93-1.04 | 0.585 |  |
|  |  |  | MR-PRESSO test | 11 | 0.99 | 0.97-1.02 | 0.574 |  |
|  |  |  | MR-Egger | 11 | 1.01 | 0.82-1.25 | 0.908 | 0.850* |
| Eisenbergiella |  |  |  |  |  |  |  |  |
|  | 4.52% | 72.37 | Inverse-variance weighted (fixed) | 12 | 0.95 | 0.91-1.00 | 0.030 | 0.602 |
|  |  |  | Weighted median | 12 | 0.92 | 0.87-0.98 | 0.007 |  |
|  |  |  | MR-PRESSO test | 12 | 0.95 | 0.91-0.99 | 0.037 |  |
|  |  |  | MR-Egger | 12 | 0.69 | 0.49-0.98 | 0.038 | 0.071* |
| Enterorhabdus |  |  |  |  |  |  |  |  |
|  | 3.56% | 67.68 | Inverse-variance weighted (fixed) | 10 | 1.03 | 0.98-1.08 | 0.294 | 0.658 |
|  |  |  | Weighted median | 10 | 1.03 | 0.96-1.11 | 0.379 |  |
|  |  |  | MR-PRESSO test | 10 | 1.03 | 0.98-1.08 | 0.258 |  |
|  |  |  | MR-Egger | 10 | 1.08 | 0.92-1.26 | 0.351 | 0.540* |
| Erysipelatoclostridium |  |  |  |  |  |  |  |  |
|  | 4.07% | 45.68 | Inverse-variance weighted (fixed) | 17 | 1.03 | 0.98-1.08 | 0.203 | 0.058 |
|  |  |  | Weighted median | 17 | 1.05 | 0.98-1.13 | 0.191 |  |
|  |  |  | MR-PRESSO test | 17 | 1.03 | 0.97-1.10 | 0.331 |  |
|  |  |  | MR-Egger | 17 | 0.96 | 0.73-1.25 | 0.738 | 0.557* |
| ErysipelotrichaceaeUCG003 |  |  |  |  |  |  |  |  |
|  | 3.28% | 34.53 | Inverse-variance weighted (fixed) | 18 | 0.96 | 0.90-1.01 | 0.110 | 0.068 |
|  |  |  | Weighted median | 18 | 0.94 | 0.86-1.02 | 0.116 |  |
|  |  |  | MR-PRESSO test | 18 | 0.96 | 0.89-1.02 | 0.217 |  |
|  |  |  | MR-Egger | 18 | 0.90 | 0.74-1.11 | 0.332 | 0.572* |
| Escherichia.Shigella |  |  |  |  |  |  |  |  |
|  | 2.97% | 37.39 | Inverse-variance weighted (fixed) | 15 | 1.03 | 0.98-1.10 | 0.258 | 0.177 |
|  |  |  | Weighted median | 15 | 1.03 | 0.95-1.11 | 0.531 |  |
|  |  |  | MR-PRESSO test | 15 | 1.03 | 0.97-1.11 | 0.344 |  |
|  |  |  | MR-Egger | 15 | 1.01 | 0.83-1.25 | 0.890 | 0.845* |
| Faecalibacterium |  |  |  |  |  |  |  |  |
|  | 2.60% | 37.59 | Inverse-variance weighted (fixed) | 13 | 1.00 | 0.94-1.07 | 0.957 | 0.461 |
|  |  |  | Weighted median | 13 | 0.98 | 0.90-1.08 | 0.738 |  |
|  |  |  | MR-PRESSO test | 13 | 1.00 | 0.94-1.07 | 0.958 |  |
|  |  |  | MR-Egger | 13 | 1.04 | 0.90-1.20 | 0.573 | 0.540* |
| FamilyXIIIAD3011 (ID: 11293) |  |  |  |  |  |  |  |  |
|  | 2.43% | 30.37 | Inverse-variance weighted (fixed) | 15 | 1.05 | 0.98-1.12 | 0.174 | 0.068 |
|  |  |  | Weighted median | 15 | 1.03 | 0.95-1.13 | 0.460 |  |
|  |  |  | MR-PRESSO test | 15 | 1.05 | 0.96-1.14 | 0.302 |  |
|  |  |  | MR-Egger | 15 | 1.21 | 0.80-1.84 | 0.363 | 0.476* |
| FamilyXIIIUCG001 (ID: 11294) |  |  |  |  |  |  |  |  |
|  | 1.94% | 36.25 | Inverse-variance weighted (fixed) | 10 | 1.00 | 0.93-1.08 | 0.967 | 0.116 |
|  |  |  | Weighted median | 10 | 1.01 | 0.91-1.12 | 0.862 |  |
|  |  |  | MR-PRESSO test | 10 | 1.00 | 0.91-1.10 | 0.974 |  |
|  |  |  | MR-Egger | 10 | 1.05 | 0.80-1.38 | 0.734 | 0.725* |
| Flavonifractor |  |  |  |  |  |  |  |  |
|  | 2.19% | 40.97 | Inverse-variance weighted (fixed) | 10 | 1.10 | 1.03-1.18 | 0.005 | 0.696 |
|  |  |  | Weighted median | 10 | 1.13 | 1.03-1.24 | 0.009 |  |
|  |  |  | MR-PRESSO test | 10 | 1.10 | 1.04-1.17 | 0.008 |  |
|  |  |  | MR-Egger | 10 | 1.29 | 0.98-1.69 | 0.070 | 0.252* |
| Fusicatenibacter |  |  |  |  |  |  |  |  |
|  | 2.60% | 24.45 | Inverse-variance weighted (fixed) | 20 | 1.01 | 0.95-1.07 | 0.856 | 0.081 |
|  |  |  | Weighted median | 20 | 0.99 | 0.91-1.09 | 0.906 |  |
|  |  |  | MR-PRESSO test | 20 | 1.01 | 0.93-1.08 | 0.883 |  |
|  |  |  | MR-Egger | 20 | 0.72 | 0.56-0.93 | 0.011 | 0.008* |
| Gordonibacter |  |  |  |  |  |  |  |  |
|  | 9.26% | 124.67 | Inverse-variance weighted (fixed) | 15 | 1.02 | 0.98-1.05 | 0.325 | 0.353 |
|  |  |  | Weighted median | 15 | 1.00 | 0.96-1.05 | 0.909 |  |
|  |  |  | MR-PRESSO test | 15 | 1.02 | 0.98-1.05 | 0.364 |  |
|  |  |  | MR-Egger | 15 | 1.03 | 0.89-1.19 | 0.732 | 0.904* |
| Haemophilus |  |  |  |  |  |  |  |  |
|  | 4.10% | 55.89 | Inverse-variance weighted (fixed) | 14 | 0.97 | 0.93-1.02 | 0.244 | 0.071 |
|  |  |  | Weighted median | 14 | 0.98 | 0.92-1.05 | 0.623 |  |
|  |  |  | MR-PRESSO test | 14 | 0.97 | 0.83-1.14 | 0.741 |  |
|  |  |  | MR-Egger | 14 | 0.97 | 0.92-1.03 | 0.377 | 0.984* |
| Holdemanella |  |  |  |  |  |  |  |  |
|  | 4.79% | 65.87 | Inverse-variance weighted (random) | 14 | 1.00 | 0.94-1.07 | 0.922 | 0.018 |
|  |  |  | Weighted median | 14 | 0.99 | 0.93-1.06 | 0.859 |  |
|  |  |  | MR-PRESSO test | 14 | 1.00 | 0.94-1.07 | 0.923 |  |
|  |  |  | Outlier corrected (MR-PRESSO) | 13 | 1.02 | 0.97-1.08 | 0.371 |  |
|  |  |  | MR-Egger | 14 | 1.07 | 0.86-1.33 | 0.566 | 0.568* |
| Holdemania |  |  |  |  |  |  |  |  |
|  | 4.84% | 51.77 | Inverse-variance weighted (fixed) | 18 | 0.97 | 0.93-1.02 | 0.196 | 0.336 |
|  |  |  | Weighted median | 18 | 0.96 | 0.90-1.03 | 0.287 |  |
|  |  |  | MR-PRESSO test | 18 | 0.97 | 0.93-1.02 | 0.236 |  |
|  |  |  | MR-Egger | 18 | 0.95 | 0.82-1.09 | 0.431 | 0.690* |
| Howardella |  |  |  |  |  |  |  |  |
|  | 6.28% | 111.64 | Inverse-variance weighted (fixed) | 11 | 1.02 | 0.99-1.06 | 0.218 | 0.758 |
|  |  |  | Weighted median | 11 | 1.01 | 0.96-1.06 | 0.820 |  |
|  |  |  | MR-PRESSO test | 11 | 1.02 | 0.99-1.06 | 0.161 |  |
|  |  |  | MR-Egger | 11 | 0.92 | 0.77-1.10 | 0.339 | 0.212* |
| Hungatella |  |  |  |  |  |  |  |  |
|  | 2.50% | 93.98 | Inverse-variance weighted (fixed) | 5 | 1.00 | 0.94-1.06 | 0.885 | 0.085 |
|  |  |  | Weighted median | 5 | 0.98 | 0.90-1.06 | 0.583 |  |
|  |  |  | MR-PRESSO test | 5 | 1.00 | 0.91-1.09 | 0.925 |  |
|  |  |  | MR-Egger | 5 | 1.20 | 0.68-2.14 | 0.530 | 0.514* |
| Intestinibacter |  |  |  |  |  |  |  |  |
|  | 2.71% | 33.98 | Inverse-variance weighted (fixed) | 15 | 1.02 | 0.96-1.08 | 0.594 | 0.316 |
|  |  |  | Weighted median | 15 | 0.99 | 0.91-1.08 | 0.833 |  |
|  |  |  | MR-PRESSO test | 15 | 1.02 | 0.96-1.08 | 0.626 |  |
|  |  |  | MR-Egger | 15 | 1.03 | 0.84-1.28 | 0.760 | 0.866* |
| Intestinimonas |  |  |  |  |  |  |  |  |
|  | 4.41% | 42.28 | Inverse-variance weighted (random) | 20 | 1.00 | 0.94-1.07 | 0.938 | 0.031 |
|  |  |  | Weighted median | 20 | 1.03 | 0.96-1.11 | 0.386 |  |
|  |  |  | MR-PRESSO test | 19 | 1.00 | 0.94-1.07 | 0.939 |  |
|  |  |  | Outlier corrected (MR-PRESSO) | 19 | 1.02 | 0.97-1.08 | 0.462 |  |
|  |  |  | MR-Egger | 20 | 1.02 | 0.86-1.19 | 0.851 | 0.864* |
| Lachnoclostridium |  |  |  |  |  |  |  |  |
|  | 1.86% | 23.18 | Inverse-variance weighted (random) | 15 | 1.01 | 0.90-1.14 | 0.822 | 0.001 |
|  |  |  | Weighted median | 15 | 0.96 | 0.86-1.07 | 0.484 |  |
|  |  |  | MR-PRESSO test | 14 | 1.01 | 0.90-1.14 | 0.825 |  |
|  |  |  | Outlier corrected (MR-PRESSO) | 14 | 0.97 | 0.89-1.05 | 0.460 |  |
|  |  |  | MR-Egger | 15 | 1.11 | 0.72-1.71 | 0.648 | 0.681* |
| Lachnospira |  |  |  |  |  |  |  |  |
|  | 0.95% | 25.08 | Inverse-variance weighted (fixed) | 7 | 0.97 | 0.87-1.07 | 0.501 | 0.468 |
|  |  |  | Weighted median | 7 | 1.01 | 0.88-1.16 | 0.894 |  |
|  |  |  | MR-PRESSO test | 7 | 0.97 | 0.88-1.06 | 0.513 |  |
|  |  |  | MR-Egger | 7 | 1.17 | 0.65-2.08 | 0.601 | 0.515* |
| LachnospiraceaeFCS020 |  |  |  |  |  |  |  |  |
|  | 3.11% | 34.54 | Inverse-variance weighted (random) | 17 | 1.02 | 0.94-1.10 | 0.665 | 0.015 |
|  |  |  | Weighted median | 17 | 1.07 | 0.99-1.16 | 0.107 |  |
|  |  |  | MR-PRESSO test | 16 | 1.02 | 0.94-1.10 | 0.671 |  |
|  |  |  | MR-Egger | 17 | 1.02 | 0.83-1.25 | 0.861 | 0.988* |
| LachnospiraceaeNC2004 |  |  |  |  |  |  |  |  |
|  | 3.49% | 66.37 | Inverse-variance weighted (fixed) | 10 | 1.01 | 0.95-1.06 | 0.826 | 0.352 |
|  |  |  | Weighted median | 10 | 1.00 | 0.94-1.08 | 0.919 |  |
|  |  |  | MR-PRESSO test | 10 | 1.01 | 0.95-1.06 | 0.840 |  |
|  |  |  | MR-Egger | 10 | 1.05 | 0.82-1.34 | 0.723 | 0.751* |
| LachnospiraceaeND3007 |  |  |  |  |  |  |  |  |
|  | 0.57% | 26.17 | Inverse-variance weighted (fixed) | 4 | 1.00 | 0.87-1.14 | 0.985 | 0.264 |
|  |  |  | Weighted median | 4 | 0.96 | 0.81-1.13 | 0.607 |  |
|  |  |  | MR-PRESSO test | 4 | 1.00 | 0.86-1.17 | 0.988 |  |
|  |  |  | MR-Egger | 4 | 0.36 | 0.02-5.31 | 0.460 | 0.460* |
| LachnospiraceaeNK4A136 |  |  |  |  |  |  |  |  |
|  | 2.75% | 32.32 | Inverse-variance weighted (fixed) | 16 | 1.01 | 0.95-1.07 | 0.700 | 0.315 |
|  |  |  | Weighted median | 16 | 1.02 | 0.94-1.12 | 0.571 |  |
|  |  |  | MR-PRESSO test | 16 | 1.01 | 0.95-1.08 | 0.723 |  |
|  |  |  | MR-Egger | 16 | 0.94 | 0.83-1.07 | 0.351 | 0.209 |
| LachnospiraceaeUCG001 |  |  |  |  |  |  |  |  |
|  | 3.66% | 43.55 | Inverse-variance weighted (fixed) | 16 | 0.99 | 0.94-1.04 | 0.700 | 0.373 |
|  |  |  | Weighted median | 16 | 0.99 | 0.92-1.07 | 0.837 |  |
|  |  |  | MR-PRESSO test | 16 | 0.99 | 0.94-1.04 | 0.715 |  |
|  |  |  | MR-Egger | 16 | 1.03 | 0.79-1.34 | 0.819 | 0.755* |
| LachnospiraceaeUCG004 |  |  |  |  |  |  |  |  |
|  | 2.28% | 28.45 | Inverse-variance weighted (fixed) | 15 | 1.00 | 0.94-1.07 | 0.951 | 0.267 |
|  |  |  | Weighted median | 15 | 0.99 | 0.90-1.08 | 0.792 |  |
|  |  |  | MR-PRESSO test | 15 | 1.00 | 0.93-1.07 | 0.956 |  |
|  |  |  | MR-Egger | 15 | 0.92 | 0.69-1.23 | 0.583 | 0.561* |
| LachnospiraceaeUCG008 |  |  |  |  |  |  |  |  |
|  | 4.84% | 66.62 | Inverse-variance weighted (fixed) | 14 | 1.00 | 0.95-1.04 | 0.929 | 0.823 |
|  |  |  | Weighted median | 14 | 1.00 | 0.95-1.06 | 0.899 |  |
|  |  |  | MR-PRESSO test | 14 | 1.00 | 0.96-1.03 | 0.913 |  |
|  |  |  | MR-Egger | 14 | 0.95 | 0.75-1.20 | 0.659 | 0.665* |
| LachnospiraceaeUCG010 |  |  |  |  |  |  |  |  |
|  | 2.52% | 36.40 | Inverse-variance weighted (fixed) | 13 | 0.96 | 0.90-1.02 | 0.179 | 0.788 |
|  |  |  | Weighted median | 13 | 0.97 | 0.89-1.06 | 0.533 |  |
|  |  |  | MR-PRESSO test | 13 | 0.96 | 0.91-1.01 | 0.125 |  |
|  |  |  | MR-Egger | 13 | 1.12 | 0.94-1.33 | 0.195 | 0.056* |
| Lactobacillus |  |  |  |  |  |  |  |  |
|  | 4.16% | 66.24 | Inverse-variance weighted (fixed) | 12 | 0.96 | 0.92-1.01 | 0.127 | 0.327 |
|  |  |  | Weighted median | 12 | 0.96 | 0.90-1.03 | 0.236 |  |
|  |  |  | MR-PRESSO test | 12 | 0.96 | 0.92-1.01 | 0.180 |  |
|  |  |  | MR-Egger | 12 | 1.00 | 0.85-1.18 | 0.987 | 0.655* |
| Lactococcus |  |  |  |  |  |  |  |  |
|  | 5.95% | 105.41 | Inverse-variance weighted (random) | 11 | 1.02 | 0.97-1.08 | 0.490 | 0.032 |
|  |  |  | Weighted median | 11 | 1.03 | 0.98-1.09 | 0.272 |  |
|  |  |  | MR-PRESSO test | 9 | 1.02 | 0.97-1.08 | 0.505 |  |
|  |  |  | Outlier corrected (MR-PRESSO) | 9 | 1.02 | 0.99-1.04 | 0.299 |  |
|  |  |  | MR-Egger | 11 | 0.89 | 0.68-1.16 | 0.377 | 0.295* |
| Marvinbryantia |  |  |  |  |  |  |  |  |
|  | 2.43% | 35.13 | Inverse-variance weighted (fixed) | 13 | 0.99 | 0.92-1.05 | 0.648 | 0.965 |
|  |  |  | Weighted median | 13 | 0.99 | 0.92-1.08 | 0.899 |  |
|  |  |  | MR-PRESSO test | 13 | 0.99 | 0.95-1.03 | 0.484 |  |
|  |  |  | MR-Egger | 13 | 0.87 | 0.68-1.10 | 0.234 | 0.268* |
| Methanobrevibacter |  |  |  |  |  |  |  |  |
|  | 4.61% | 110.62 | Inverse-variance weighted (fixed) | 8 | 0.97 | 0.93-1.02 | 0.227 | 0.529 |
|  |  |  | Weighted median | 8 | 0.97 | 0.92-1.03 | 0.283 |  |
|  |  |  | MR-PRESSO test | 8 | 0.97 | 0.93-1.01 | 0.237 |  |
|  |  |  | MR-Egger | 8 | 1.10 | 0.93-1.29 | 0.288 | 0.153* |
| Odoribacter |  |  |  |  |  |  |  |  |
|  | 1.45% | 30.01 | Inverse-variance weighted (fixed) | 9 | 0.97 | 0.89-1.05 | 0.463 | 0.197 |
|  |  |  | Weighted median | 9 | 1.01 | 0.90-1.13 | 0.862 |  |
|  |  |  | MR-PRESSO test | 9 | 0.97 | 0.88-1.07 | 0.551 |  |
|  |  |  | MR-Egger | 9 | 1.26 | 0.94-1.69 | 0.129 | 0.072* |
| Olsenella |  |  |  |  |  |  |  |  |
|  | 6.41% | 114.06 | Inverse-variance weighted (fixed) | 11 | 0.98 | 0.94-1.02 | 0.265 | 0.275 |
|  |  |  | Weighted median | 11 | 0.96 | 0.91-1.02 | 0.162 |  |
|  |  |  | MR-PRESSO test | 11 | 0.98 | 0.94-1.02 | 0.335 |  |
|  |  |  | MR-Egger | 11 | 1.09 | 0.93-1.27 | 0.275 | 0.155* |
| Oscillibacter |  |  |  |  |  |  |  |  |
|  | 4.56% | 51.50 | Inverse-variance weighted (random) | 17 | 1.01 | 0.95-1.07 | 0.802 | 0.043 |
|  |  |  | Weighted median | 17 | 1.02 | 0.95-1.09 | 0.559 |  |
|  |  |  | MR-PRESSO test | 17 | 1.01 | 0.95-1.07 | 0.805 |  |
|  |  |  | MR-Egger | 17 | 1.01 | 0.81-1.26 | 0.919 | 0.971* |
| Oscillospira |  |  |  |  |  |  |  |  |
|  | 2.17% | 40.72 | Inverse-variance weighted (fixed) | 10 | 0.98 | 0.91-1.04 | 0.448 | 0.087 |
|  |  |  | Weighted median | 10 | 0.98 | 0.89-1.07 | 0.638 |  |
|  |  |  | MR-PRESSO test | 10 | 0.98 | 0.90-1.06 | 0.573 |  |
|  |  |  | MR-Egger | 10 | 1.39 | 1.03-1.87 | 0.029 | 0.016* |
| Oxalobacter |  |  |  |  |  |  |  |  |
|  | 6.51% | 106.27 | Inverse-variance weighted (fixed) | 12 | 0.98 | 0.94-1.02 | 0.382 | 0.412 |
|  |  |  | Weighted median | 12 | 0.98 | 0.93-1.03 | 0.362 |  |
|  |  |  | MR-PRESSO test | 12 | 0.98 | 0.94-1.02 | 0.408 |  |
|  |  |  | MR-Egger | 12 | 1.01 | 0.84-1.21 | 0.909 | 0.755* |
| Parabacteroides |  |  |  |  |  |  |  |  |
|  | 1.47% | 27.38 | Inverse-variance weighted (fixed) | 10 | 0.97 | 0.89-1.05 | 0.454 | 0.975 |
|  |  |  | Weighted median | 10 | 0.99 | 0.89-1.10 | 0.855 |  |
|  |  |  | MR-PRESSO test | 10 | 0.97 | 0.93-1.01 | 0.204 |  |
|  |  |  | MR-Egger | 10 | 1.10 | 0.89-1.37 | 0.391 | 0.221* |
| Paraprevotella |  |  |  |  |  |  |  |  |
|  | 4.40% | 64.84 | Inverse-variance weighted (fixed) | 13 | 0.97 | 0.93-1.02 | 0.216 | 0.716 |
|  |  |  | Weighted median | 13 | 0.97 | 0.91-1.04 | 0.381 |  |
|  |  |  | MR-PRESSO test | 13 | 0.97 | 0.93-1.01 | 0.175 |  |
|  |  |  | MR-Egger | 13 | 0.96 | 0.82-1.11 | 0.551 | 0.817* |
| Parasutterella |  |  |  |  |  |  |  |  |
|  | 3.69% | 41.32 | Inverse-variance weighted (fixed) | 17 | 1.00 | 0.96-1.06 | 0.863 | 0.976 |
|  |  |  | Weighted median | 17 | 1.01 | 0.94-1.07 | 0.858 |  |
|  |  |  | MR-PRESSO test | 17 | 1.00 | 0.97-1.04 | 0.796 |  |
|  |  |  | MR-Egger | 17 | 1.05 | 0.90-1.23 | 0.542 | 0.558* |
| Peptococcus |  |  |  |  |  |  |  |  |
|  | 7.02% | 81.41 | Inverse-variance weighted (fixed) | 17 | 0.98 | 0.95-1.02 | 0.318 | 0.984 |
|  |  |  | Weighted median | 17 | 0.98 | 0.93-1.02 | 0.350 |  |
|  |  |  | MR-PRESSO test | 17 | 0.98 | 0.96-1.00 | 0.132 |  |
|  |  |  | MR-Egger | 17 | 0.90 | 0.78-1.03 | 0.124 | 0.188* |
| Phascolarctobacterium |  |  |  |  |  |  |  |  |
|  | 2.71% | 39.22 | Inverse-variance weighted (fixed) | 13 | 0.98 | 0.92-1.04 | 0.509 | 0.833 |
|  |  |  | Weighted median | 13 | 0.98 | 0.90-1.06 | 0.611 |  |
|  |  |  | MR-PRESSO test | 13 | 0.98 | 0.94-1.03 | 0.415 |  |
|  |  |  | MR-Egger | 13 | 0.86 | 0.69-1.08 | 0.209 | 0.262* |
| Prevotella7 |  |  |  |  |  |  |  |  |
|  | 7.34% | 121.04 | Inverse-variance weighted (fixed) | 12 | 1.00 | 0.97-1.04 | 0.923 | 0.982 |
|  |  |  | Weighted median | 12 | 0.99 | 0.95-1.04 | 0.763 |  |
|  |  |  | MR-PRESSO test | 12 | 1.00 | 0.98-1.02 | 0.868 |  |
|  |  |  | MR-Egger | 12 | 0.93 | 0.74-1.18 | 0.561 | 0.546* |
| Prevotella9 | 5.00% | 48.25 |  |  |  |  |  |  |
|  |  |  | Inverse-variance weighted (fixed) | 20 | 1.06 | 1.02-1.11 | 0.006 | 0.602 |
|  |  |  | Weighted median | 20 | 1.04 | 0.97-1.10 | 0.266 |  |
|  |  |  | MR-PRESSO test | 20 | 1.06 | 1.02-1.11 | 0.009 |  |
|  |  |  | MR-Egger | 20 | 1.16 | 1.02-1.30 | 0.018 | 0.148* |
| RikenellaceaeRC9 |  |  |  |  |  |  |  |  |
|  | 9.72% | 131.46 | Inverse-variance weighted (fixed) | 15 | 1.02 | 0.99-1.05 | 0.186 | 0.187 |
|  |  |  | Weighted median | 15 | 1.02 | 0.97-1.07 | 0.400 |  |
|  |  |  | MR-PRESSO test | 15 | 1.02 | 0.99-1.06 | 0.268 |  |
|  |  |  | MR-Egger | 15 | 0.97 | 0.77-1.23 | 0.823 | 0.684* |
| Romboutsia |  |  |  |  |  |  |  |  |
|  | 2.87% | 36.08 | Inverse-variance weighted (fixed) | 15 | 0.98 | 0.92-1.04 | 0.437 | 0.779 |
|  |  |  | Weighted median | 15 | 0.96 | 0.88-1.04 | 0.307 |  |
|  |  |  | MR-PRESSO test | 15 | 0.98 | 0.93-1.03 | 0.368 |  |
|  |  |  | MR-Egger | 15 | 0.86 | 0.73-1.00 | 0.047 | 0.067* |
| Roseburia |  |  |  |  |  |  |  |  |
|  | 2.57% | 26.88 | Inverse-variance weighted (random) | 18 | 0.97 | 0.89-1.06 | 0.533 | 0.004 |
|  |  |  | Weighted median | 18 | 0.95 | 0.87-1.04 | 0.306 |  |
|  |  |  | MR-PRESSO test | 17 | 0.97 | 0.89-1.06 | 0.541 |  |
|  |  |  | Outlier corrected (MR-PRESSO) | 17 | 0.95 | 0.88-1.03 | 0.255 |  |
|  |  |  | MR-Egger | 18 | 1.02 | 0.78-1.32 | 0.910 | 0.724* |
| Ruminiclostridium5 |  |  |  |  |  |  |  |  |
|  | 2.06% | 25.73 | Inverse-variance weighted (fixed) | 15 | 1.05 | 0.98-1.13 | 0.140 | 0.247 |
|  |  |  | Weighted median | 15 | 1.10 | 1.00-1.22 | 0.061 |  |
|  |  |  | MR-PRESSO test | 15 | 1.05 | 0.98-1.14 | 0.204 |  |
|  |  |  | MR-Egger | 15 | 0.95 | 0.74-1.22 | 0.685 | 0.394* |
| Ruminiclostridium6 |  |  |  |  |  |  |  |  |
|  | 3.28% | 36.50 | Inverse-variance weighted (fixed) | 17 | 1.06 | 1.00-1.12 | 0.046 | 0.268 |
|  |  |  | Weighted median | 17 | 1.06 | 0.98-1.14 | 0.173 |  |
|  |  |  | MR-PRESSO test | 17 | 1.06 | 1.00-1.12 | 0.086 |  |
|  |  |  | MR-Egger | 17 | 0.99 | 0.85-1.15 | 0.867 | 0.340* |
| Ruminiclostridium9 |  |  |  |  |  |  |  |  |
|  | 2.20% | 25.71 | Inverse-variance weighted (fixed) | 16 | 1.04 | 0.98-1.12 | 0.197 | 0.323 |
|  |  |  | Weighted median | 16 | 1.06 | 0.97-1.17 | 0.193 |  |
|  |  |  | MR-PRESSO test | 16 | 1.04 | 0.97-1.12 | 0.243 |  |
|  |  |  | MR-Egger | 16 | 1.08 | 0.77-1.53 | 0.645 | 0.827* |
| RuminococcaceaeNK4A214 |  |  |  |  |  |  |  |  |
|  | 2.76% | 28.93 | Inverse-variance weighted (fixed) | 18 | 1.05 | 0.99-1.11 | 0.131 | 0.480 |
|  |  |  | Weighted median | 18 | 0.99 | 0.91-1.08 | 0.842 |  |
|  |  |  | MR-PRESSO test | 18 | 1.05 | 0.99-1.11 | 0.146 |  |
|  |  |  | MR-Egger | 18 | 0.81 | 0.68-0.98 | 0.028 | 0.004* |
| RuminococcaceaeUCG002 |  |  |  |  |  |  |  |  |
|  | 3.86% | 28.25 | Inverse-variance weighted (fixed) | 26 | 1.01 | 0.96-1.06 | 0.810 | 0.281 |
|  |  |  | Weighted median | 26 | 1.00 | 0.93-1.08 | 0.977 |  |
|  |  |  | MR-PRESSO test | 26 | 1.01 | 0.95-1.06 | 0.824 |  |
|  |  |  | MR-Egger | 26 | 0.96 | 0.83-1.12 | 0.595 | 0.514* |
| RuminococcaceaeUCG003 |  |  |  |  |  |  |  |  |
|  | 2.28% | 30.60 | Inverse-variance weighted (fixed) | 14 | 1.02 | 0.95-1.08 | 0.634 | 0.056 |
|  |  |  | Weighted median | 14 | 0.96 | 0.87-1.06 | 0.406 |  |
|  |  |  | MR-PRESSO test | 14 | 1.02 | 0.93-1.10 | 0.720 |  |
|  |  |  | MR-Egger | 14 | 1.08 | 0.81-1.45 | 0.606 | 0.667* |
| RuminococcaceaeUCG004 |  |  |  |  |  |  |  |  |
|  | 2.87% | 45.19 | Inverse-variance weighted (fixed) | 12 | 1.02 | 0.96-1.09 | 0.466 | 0.840 |
|  |  |  | Weighted median | 12 | 1.02 | 0.94-1.10 | 0.698 |  |
|  |  |  | MR-PRESSO test | 12 | 1.02 | 0.98-1.07 | 0.362 |  |
|  |  |  | MR-Egger | 12 | 1.24 | 0.88-1.73 | 0.217 | 0.261* |
| RuminococcaceaeUCG005 |  |  |  |  |  |  |  |  |
|  | 2.73% | 30.20 | Inverse-variance weighted (fixed) | 17 | 1.02 | 0.96-1.08 | 0.528 | 0.117 |
|  |  |  | Weighted median | 17 | 1.05 | 0.96-1.14 | 0.275 |  |
|  |  |  | MR-PRESSO test | 17 | 1.02 | 0.95-1.09 | 0.605 |  |
|  |  |  | MR-Egger | 17 | 1.04 | 0.85-1.28 | 0.708 | 0.834* |
| RuminococcaceaeUCG009 |  |  |  |  |  |  |  |  |
|  | 4.30% | 58.81 | Inverse-variance weighted (random) | 14 | 0.96 | 0.89-1.03 | 0.256 | 0.005 |
|  |  |  | Weighted median | 14 | 0.95 | 0.88-1.02 | 0.165 |  |
|  |  |  | MR-PRESSO test | 13 | 0.96 | 0.89-1.03 | 0.276 |  |
|  |  |  | Outlier corrected (MR-PRESSO) | 13 | 0.93 | 0.88-1.00 | 0.065 |  |
|  |  |  | MR-Egger | 14 | 1.34 | 1.04-1.73 | 0.024 | 0.008* |
| RuminococcaceaeUCG010 |  |  |  |  |  |  |  |  |
|  | 1.51% | 35.03 | Inverse-variance weighted (fixed) | 8 | 0.98 | 0.91-1.06 | 0.662 | 0.574 |
|  |  |  | Weighted median | 8 | 0.99 | 0.89-1.10 | 0.819 |  |
|  |  |  | MR-PRESSO test | 8 | 0.98 | 0.91-1.06 | 0.643 |  |
|  |  |  | MR-Egger | 8 | 1.18 | 0.86-1.62 | 0.307 | 0.240* |
| RuminococcaceaeUCG011 |  |  |  |  |  |  |  |  |
|  | 4.90% | 118.09 | Inverse-variance weighted (fixed) | 8 | 1.01 | 0.97-1.06 | 0.635 | 0.759 |
|  |  |  | Weighted median | 8 | 1.01 | 0.96-1.07 | 0.591 |  |
|  |  |  | MR-PRESSO test | 8 | 1.01 | 0.98-1.04 | 0.558 |  |
|  |  |  | MR-Egger | 8 | 1.01 | 0.80-1.26 | 0.948 | 0.979* |
| RuminococcaceaeUCG013 |  |  |  |  |  |  |  |  |
|  | 2.09% | 26.01 | Inverse-variance weighted (fixed) | 15 | 0.99 | 0.93-1.06 | 0.735 | 0.245 |
|  |  |  | Weighted median | 15 | 0.98 | 0.89-1.08 | 0.685 |  |
|  |  |  | MR-PRESSO test | 15 | 0.99 | 0.92-1.06 | 0.765 |  |
|  |  |  | MR-Egger | 15 | 0.81 | 0.68-0.97 | 0.024 | 0.022* |
| RuminococcaceaeUCG014 |  |  |  |  |  |  |  |  |
|  | 3.02% | 31.68 | Inverse-variance weighted (fixed) | 18 | 1.05 | 0.99-1.11 | 0.101 | 0.079 |
|  |  |  | Weighted median | 18 | 1.04 | 0.96-1.13 | 0.369 |  |
|  |  |  | MR-PRESSO test | 18 | 1.05 | 0.98-1.13 | 0.201 |  |
|  |  |  | MR-Egger | 18 | 1.10 | 0.88-1.37 | 0.410 | 0.671* |
| Ruminococcus1 |  |  |  |  |  |  |  |  |
|  | 2.14% | 28.60 | Inverse-variance weighted (fixed) | 14 | 0.96 | 0.90-1.03 | 0.299 | 0.657 |
|  |  |  | Weighted median | 14 | 0.95 | 0.87-1.04 | 0.298 |  |
|  |  |  | MR-PRESSO test | 14 | 0.96 | 0.91-1.03 | 0.268 |  |
|  |  |  | MR-Egger | 14 | 0.95 | 0.78-1.16 | 0.623 | 0.880* |
| Ruminococcus2 |  |  |  |  |  |  |  |  |
|  | 2.76% | 34.63 | Inverse-variance weighted (random) | 15 | 1.01 | 0.93-1.11 | 0.766 | 0.019 |
|  |  |  | Weighted median | 15 | 0.99 | 0.90-1.09 | 0.820 |  |
|  |  |  | MR-PRESSO test | 14 | 1.01 | 0.93-1.11 | 0.770 |  |
|  |  |  | Outlier corrected (MR-PRESSO) | 14 | 1.04 | 0.96-1.12 | 0.370 |  |
|  |  |  | MR-Egger | 15 | 0.90 | 0.71-1.14 | 0.363 | 0.271* |
| Sellimonas |  |  |  |  |  |  |  |  |
|  | 9.32% | 144.84 | Inverse-variance weighted (fixed) | 13 | 1.00 | 0.97-1.03 | 0.849 | 0.074 |
|  |  |  | Weighted median | 13 | 1.00 | 0.95-1.05 | 0.987 |  |
|  |  |  | MR-PRESSO test | 13 | 1.00 | 0.96-1.04 | 0.885 |  |
|  |  |  | MR-Egger | 13 | 1.01 | 0.81-1.26 | 0.929 | 0.904* |
| Senegalimassilia |  |  |  |  |  |  |  |  |
|  | 2.58% | 60.59 | Inverse-variance weighted (fixed) | 8 | 0.99 | 0.93-1.05 | 0.684 | 0.610 |
|  |  |  | Weighted median | 8 | 0.99 | 0.92-1.07 | 0.850 |  |
|  |  |  | MR-PRESSO test | 8 | 0.99 | 0.94-1.04 | 0.657 |  |
|  |  |  | MR-Egger | 8 | 1.00 | 0.78-1.28 | 0.990 | 0.927* |
| Slackia |  |  |  |  |  |  |  |  |
|  | 3.10% | 65.21 | Inverse-variance weighted (fixed) | 9 | 0.96 | 0.91-1.01 | 0.105 | 0.340 |
|  |  |  | Weighted median | 9 | 0.94 | 0.87-1.01 | 0.085 |  |
|  |  |  | MR-PRESSO test | 9 | 0.96 | 0.90-1.01 | 0.166 |  |
|  |  |  | MR-Egger | 9 | 0.96 | 0.74-1.25 | 0.776 | 0.960* |
| Streptococcus |  |  |  |  |  |  |  |  |
|  | 2.68% | 26.56 | Inverse-variance weighted (fixed) | 19 | 0.95 | 0.90-1.01 | 0.110 | 0.276 |
|  |  |  | Weighted median | 19 | 1.00 | 0.91-1.09 | 0.927 |  |
|  |  |  | MR-PRESSO test | 19 | 0.95 | 0.89-1.02 | 0.157 |  |
|  |  |  | MR-Egger | 19 | 1.10 | 0.86-1.40 | 0.458 | 0.241* |
| Subdoligranulum |  |  |  |  |  |  |  |  |
|  | 1.86% | 24.81 | Inverse-variance weighted (fixed) | 14 | 1.01 | 0.94-1.08 | 0.786 | 0.328 |
|  |  |  | Weighted median | 14 | 1.04 | 0.94-1.15 | 0.421 |  |
|  |  |  | MR-PRESSO test | 14 | 1.01 | 0.94-1.09 | 0.803 |  |
|  |  |  | MR-Egger | 14 | 1.00 | 0.81-1.25 | 0.984 | 0.943* |
| Sutterella |  |  |  |  |  |  |  |  |
|  | 1.97% | 30.70 | Inverse-variance weighted (fixed) | 12 | 1.03 | 0.96-1.10 | 0.419 | 0.565 |
|  |  |  | Weighted median | 12 | 1.02 | 0.93-1.12 | 0.622 |  |
|  |  |  | MR-PRESSO test | 12 | 1.03 | 0.97-1.10 | 0.406 |  |
|  |  |  | MR-Egger | 12 | 1.19 | 0.86-1.65 | 0.289 | 0.361* |
| Terrisporobacter |  |  |  |  |  |  |  |  |
|  | 2.14% | 66.94 | Inverse-variance weighted (fixed) | 6 | 0.98 | 0.92-1.05 | 0.624 | 0.855 |
|  |  |  | Weighted median | 6 | 0.96 | 0.89-1.04 | 0.323 |  |
|  |  |  | MR-PRESSO test | 6 | 0.98 | 0.94-1.02 | 0.469 |  |
|  |  |  | MR-Egger | 6 | 0.97 | 0.80-1.17 | 0.752 | 0.876* |
| Turicibacter |  |  |  |  |  |  |  |  |
|  | 3.35% | 45.43 | Inverse-variance weighted (fixed) | 14 | 0.98 | 0.93-1.03 | 0.490 | 0.436 |
|  |  |  | Weighted median | 14 | 0.98 | 0.92-1.05 | 0.646 |  |
|  |  |  | MR-PRESSO test | 14 | 0.98 | 0.93-1.03 | 0.504 |  |
|  |  |  | MR-Egger | 14 | 0.91 | 0.73-1.14 | 0.423 | 0.510* |
| Tyzzerella3 |  |  |  |  |  |  |  |  |
|  | 6.44% | 90.12 | Inverse-variance weighted (random) | 14 | 1.00 | 0.94-1.05 | 0.853 | 0.045 |
|  |  |  | Weighted median | 14 | 1.01 | 0.95-1.06 | 0.863 |  |
|  |  |  | MR-PRESSO test | 14 | 1.00 | 0.94-1.05 | 0.856 |  |
|  |  |  | Outlier corrected (MR-PRESSO) | 13 | 1.01 | 0.97-1.06 | 0.527 |  |
|  |  |  | MR-Egger | 14 | 0.92 | 0.68-1.24 | 0.594 | 0.611* |
| Veillonella |  |  |  |  |  |  |  |  |
|  | 2.77% | 47.44 | Inverse-variance weighted (fixed) | 11 | 0.98 | 0.92-1.04 | 0.469 | 0.468 |
|  |  |  | Weighted median | 11 | 0.98 | 0.90-1.06 | 0.544 |  |
|  |  |  | MR-PRESSO test | 11 | 0.98 | 0.92-1.04 | 0.479 |  |
|  |  |  | MR-Egger | 11 | 0.88 | 0.61-1.27 | 0.490 | 0.561* |
| Victivallis |  |  |  |  |  |  |  |  |
|  | 8.61% | 132.74 | Inverse-variance weighted (fixed) | 13 | 1.00 | 0.97-1.03 | 0.944 | 0.105 |
|  |  |  | Weighted median | 13 | 1.03 | 0.98-1.08 | 0.291 |  |
|  |  |  | MR-PRESSO test | 13 | 1.00 | 0.96-1.04 | 0.956 |  |
|  |  |  | MR-Egger | 13 | 0.92 | 0.71-1.19 | 0.515 | 0.516* |

^1^ CI, confidence interval; IBS, irritable bowel syndrome; MR, Mendelian randomization; MR-PRESSO test, MR Pleiotropy RESidual Sum and Outlier test; OR, odds ratio; SNP, single nucleotide polymorphism. **P*-value of the intercept from MR-Egger regression.

**Supplementary Table 2** Characteristics of the genetic variants associated with 11 bacterial that have been identified to be associated with the risk of IBS^1^

| Gut microbiota | SNP | Chr | Position | Effect allele | Beta | SE | *P*-value |
| --- | --- | --- | --- | --- | --- | --- | --- |
| Phylum Actinobacteria | rs1879087 | 2 | 136822223 | C | 0.102 | 0.022 | 2.48E-07 |
| Phylum Actinobacteria | rs4429415 | 2 | 213756191 | C | 0.058 | 0.011 | 2.05E-07 |
| Phylum Actinobacteria | rs6430601 | 2 | 136854537 | T | 0.070 | 0.015 | 2.38E-06 |
| Phylum Actinobacteria | rs6743026 | 2 | 102249583 | T | 0.059 | 0.013 | 9.88E-06 |
| Phylum Actinobacteria | rs7570971 | 2 | 135837906 | A | 0.087 | 0.011 | 1.41E-14 |
| Phylum Actinobacteria | rs80124826 | 2 | 239936384 | T | -0.124 | 0.028 | 8.75E-06 |
| Phylum Actinobacteria | rs9833771 | 3 | 32362998 | T | 0.049 | 0.011 | 4.07E-06 |
| Phylum Actinobacteria | rs55888705 | 4 | 1517826 | A | 0.053 | 0.011 | 1.31E-06 |
| Phylum Actinobacteria | rs1397793 | 5 | 90471451 | G | -0.052 | 0.011 | 3.74E-06 |
| Phylum Actinobacteria | rs12528285 | 6 | 92775210 | C | 0.081 | 0.018 | 5.69E-06 |
| Phylum Actinobacteria | rs13192624 | 6 | 113129609 | T | -0.052 | 0.012 | 9.33E-06 |
| Phylum Actinobacteria | rs857444 | 6 | 14617591 | C | 0.051 | 0.011 | 3.80E-06 |
| Phylum Actinobacteria | rs11766971 | 7 | 155002747 | T | 0.048 | 0.011 | 9.40E-06 |
| Phylum Actinobacteria | rs62448869 | 7 | 24464799 | T | -0.052 | 0.011 | 1.14E-06 |
| Phylum Actinobacteria | rs10841473 | 12 | 20378911 | G | -0.060 | 0.012 | 4.47E-07 |
| Phylum Actinobacteria | rs74037001 | 14 | 23847194 | G | -0.082 | 0.017 | 6.71E-07 |
| Phylum Actinobacteria | rs6496870 | 15 | 92467422 | T | 0.051 | 0.011 | 4.62E-06 |
| Phylum Actinobacteria | rs34284163 | 16 | 89248299 | A | -0.060 | 0.014 | 8.82E-06 |
| Phylum Actinobacteria | rs8047955 | 16 | 81776768 | A | 0.052 | 0.011 | 2.66E-06 |
| Phylum Actinobacteria | rs75211493 | 19 | 5300465 | G | 0.084 | 0.018 | 9.27E-06 |
| Class Melainabacteria | rs16851659 | 1 | 230523039 | G | -0.090 | 0.019 | 1.27E-06 |
| Class Melainabacteria | rs9864379 | 3 | 14306949 | T | -0.160 | 0.029 | 5.36E-08 |
| Class Melainabacteria | rs73074665 | 7 | 20010624 | A | 0.166 | 0.036 | 2.87E-06 |
| Class Melainabacteria | rs10738747 | 9 | 26184578 | G | 0.081 | 0.018 | 9.96E-06 |
| Class Melainabacteria | rs113884518 | 9 | 24648997 | T | -0.205 | 0.045 | 8.06E-06 |
| Class Melainabacteria | rs1221147 | 9 | 122046549 | T | 0.124 | 0.028 | 7.11E-06 |
| Class Melainabacteria | rs4129395 | 9 | 115975389 | G | 0.090 | 0.019 | 1.48E-06 |
| Class Melainabacteria | rs367480 | 11 | 2937631 | G | -0.084 | 0.019 | 8.20E-06 |
| Class Melainabacteria | rs10148250 | 14 | 107061448 | G | 0.086 | 0.019 | 8.67E-06 |
| Class Melainabacteria | rs79790072 | 15 | 100747683 | T | 0.227 | 0.049 | 3.29E-06 |
| Class Melainabacteria | rs11150282 | 16 | 80493705 | T | 0.099 | 0.020 | 6.03E-07 |
| Class Melainabacteria | rs28678345 | 17 | 53906328 | T | 0.215 | 0.047 | 6.69E-06 |
| Class Melainabacteria | rs789069 | 18 | 1008278 | A | -0.104 | 0.023 | 6.85E-06 |
| Order Gastranaerophilales | rs16851659 | 1 | 230523039 | G | -0.090 | 0.019 | 1.27E-06 |
| Order Gastranaerophilales | rs9864379 | 3 | 14306949 | T | -0.161 | 0.029 | 4.66E-08 |
| Order Gastranaerophilales | rs73074665 | 7 | 20010624 | A | 0.165 | 0.036 | 3.62E-06 |
| Order Gastranaerophilales | rs113884518 | 9 | 24648997 | T | -0.206 | 0.046 | 7.74E-06 |
| Order Gastranaerophilales | rs1221147 | 9 | 122046549 | T | 0.126 | 0.028 | 4.86E-06 |
| Order Gastranaerophilales | rs4129395 | 9 | 115975389 | G | 0.090 | 0.019 | 1.22E-06 |
| Order Gastranaerophilales | rs367480 | 11 | 2937631 | G | -0.084 | 0.019 | 7.52E-06 |
| Order Gastranaerophilales | rs79790072 | 15 | 100747683 | T | 0.226 | 0.049 | 3.54E-06 |
| Order Gastranaerophilales | rs8028558 | 15 | 62055753 | A | 0.083 | 0.019 | 9.78E-06 |
| Order Gastranaerophilales | rs11150282 | 16 | 80493705 | T | 0.098 | 0.020 | 7.36E-07 |
| Order Gastranaerophilales | rs28678345 | 17 | 53906328 | T | 0.213 | 0.047 | 8.06E-06 |
| Order Gastranaerophilales | rs789069 | 18 | 1008278 | A | -0.104 | 0.023 | 6.50E-06 |
| Order Gastranaerophilales | rs3754624 | 2 | 225633812 | C | 0.094 | 0.020 | 2.68E-06 |
| Order Gastranaerophilales | rs76784716 | 2 | 169033340 | A | 0.136 | 0.028 | 1.31E-06 |
| Order Gastranaerophilales | rs55876211 | 3 | 84471266 | C | -0.087 | 0.020 | 7.87E-06 |
| Order Gastranaerophilales | rs9813022 | 3 | 13726736 | A | -0.083 | 0.016 | 3.07E-07 |
| Order Gastranaerophilales | rs77304857 | 4 | 134248015 | C | -0.100 | 0.022 | 6.02E-06 |
| Order Gastranaerophilales | rs1035406 | 5 | 119372737 | G | -0.115 | 0.025 | 4.07E-06 |
| Order Gastranaerophilales | rs1549633 | 5 | 27945645 | A | 0.100 | 0.022 | 3.88E-06 |
| Order Gastranaerophilales | rs3730086 | 5 | 67577051 | A | 0.080 | 0.018 | 7.98E-06 |
| Order Gastranaerophilales | rs7001029 | 8 | 131958403 | C | 0.121 | 0.026 | 2.83E-06 |
| Order Gastranaerophilales | rs11591293 | 10 | 113419797 | G | 0.072 | 0.016 | 4.69E-06 |
| Order Gastranaerophilales | rs4278423 | 10 | 2670553 | T | 0.105 | 0.023 | 3.98E-06 |
| Order Gastranaerophilales | rs61933850 | 12 | 73139398 | G | 0.165 | 0.036 | 7.00E-06 |
| Order Gastranaerophilales | rs11630875 | 15 | 61775734 | T | 0.095 | 0.020 | 3.70E-06 |
| Order Gastranaerophilales | rs13336560 | 16 | 88554243 | C | -0.070 | 0.016 | 9.75E-06 |
| Order Gastranaerophilales | rs4822789 | 22 | 27173098 | G | 0.073 | 0.016 | 7.33E-06 |
| Family *Rikenellaceae* | rs67705352 | 1 | 19790706 | T | -0.055 | 0.011 | 6.58E-07 |
| Family *Rikenellaceae* | rs6744030 | 2 | 174257127 | C | 0.070 | 0.016 | 9.32E-06 |
| Family *Rikenellaceae* | rs6837275 | 4 | 187842232 | A | 0.057 | 0.012 | 1.45E-06 |
| Family *Rikenellaceae* | rs9389714 | 6 | 100244667 | C | -0.064 | 0.014 | 8.79E-06 |
| Family *Rikenellaceae* | rs67281112 | 7 | 135778919 | G | 0.064 | 0.014 | 3.63E-06 |
| Family *Rikenellaceae* | rs2447496 | 8 | 99188924 | G | -0.055 | 0.012 | 6.09E-06 |
| Family *Rikenellaceae* | rs35909684 | 8 | 144441740 | A | -0.085 | 0.019 | 7.10E-06 |
| Family *Rikenellaceae* | rs7832304 | 8 | 134444155 | T | -0.072 | 0.016 | 8.71E-06 |
| Family *Rikenellaceae* | rs10217435 | 9 | 86052655 | C | -0.088 | 0.020 | 6.51E-06 |
| Family *Rikenellaceae* | rs62532512 | 9 | 14158854 | C | -0.050 | 0.011 | 2.76E-06 |
| Family *Rikenellaceae* | rs10832801 | 11 | 17589129 | A | -0.053 | 0.012 | 7.50E-06 |
| Family *Rikenellaceae* | rs1939881 | 11 | 95304834 | G | -0.106 | 0.021 | 5.64E-07 |
| Family *Rikenellaceae* | rs9578457 | 13 | 22867356 | G | -0.141 | 0.032 | 3.99E-06 |
| Family *Rikenellaceae* | rs9603208 | 13 | 38044689 | G | 0.082 | 0.016 | 1.92E-07 |
| Family *Rikenellaceae* | rs74474130 | 14 | 90270821 | T | 0.138 | 0.030 | 3.61E-06 |
| Family *Rikenellaceae* | rs77885767 | 14 | 21548634 | C | -0.156 | 0.034 | 2.85E-06 |
| Family *Rikenellaceae* | rs4264350 | 15 | 71658642 | T | -0.053 | 0.011 | 1.35E-06 |
| Family *Rikenellaceae* | rs4783173 | 16 | 85396629 | C | 0.048 | 0.011 | 7.06E-06 |
| Family *Rikenellaceae* | rs59663348 | 18 | 4069710 | G | 0.057 | 0.013 | 6.12E-06 |
| Family *Rikenellaceae* | rs7242694 | 18 | 10338470 | C | -0.062 | 0.013 | 5.81E-06 |
| Family *Rikenellaceae* | rs2833282 | 21 | 32496710 | G | 0.071 | 0.016 | 4.31E-06 |
| Family *Rikenellaceae* | rs36021379 | 21 | 28515912 | A | -0.066 | 0.014 | 7.20E-06 |
| Family *Rikenellaceae* | rs8130320 | 21 | 40580258 | A | -0.049 | 0.011 | 4.73E-06 |
| Genus *Eubacterium hallii* group | rs10798999 | 1 | 34308917 | C | 0.060 | 0.013 | 2.61E-06 |
| Genus *Eubacterium hallii* group | rs17474256 | 1 | 104524676 | G | 0.081 | 0.018 | 9.45E-06 |
| Genus *Eubacterium hallii* group | rs138531890 | 3 | 195238086 | A | 0.153 | 0.035 | 5.43E-06 |
| Genus *Eubacterium hallii* group | rs28584818 | 3 | 64664456 | A | 0.126 | 0.027 | 4.43E-06 |
| Genus *Eubacterium hallii* group | rs6550770 | 3 | 23663416 | T | -0.198 | 0.044 | 4.82E-06 |
| Genus *Eubacterium hallii* group | rs949971 | 3 | 110283389 | T | -0.054 | 0.012 | 3.29E-06 |
| Genus *Eubacterium hallii* group | rs13116360 | 4 | 111885431 | T | 0.154 | 0.030 | 2.94E-07 |
| Genus *Eubacterium hallii* group | rs17074066 | 4 | 183709536 | T | -0.081 | 0.019 | 9.35E-06 |
| Genus *Eubacterium hallii* group | rs10808115 | 7 | 100635375 | A | -0.050 | 0.011 | 4.42E-06 |
| Genus *Eubacterium hallii* group | rs60254196 | 7 | 148856720 | A | -0.052 | 0.011 | 2.70E-06 |
| Genus *Eubacterium hallii* group | rs10501370 | 11 | 58040621 | C | -0.116 | 0.025 | 5.42E-06 |
| Genus *Eubacterium hallii* group | rs117748144 | 11 | 11771637 | T | -0.127 | 0.029 | 7.86E-06 |
| Genus *Eubacterium hallii* group | rs78056098 | 11 | 123789877 | G | -0.051 | 0.011 | 8.29E-06 |
| Genus *Eubacterium hallii* group | rs74018587 | 15 | 62014160 | C | 0.209 | 0.044 | 3.70E-06 |
| Genus *Eubacterium hallii* group | rs630939 | 18 | 48384463 | C | -0.051 | 0.011 | 9.16E-06 |
| Genus *Eubacterium hallii* group | rs281379 | 19 | 49214274 | A | -0.050 | 0.011 | 9.33E-06 |
| Genus *Coprococcus* 1 | rs1010560 | 1 | 30400301 | C | 0.058 | 0.012 | 1.96E-06 |
| Genus *Coprococcus* 1 | rs74101919 | 1 | 95424321 | T | -0.072 | 0.014 | 1.03E-06 |
| Genus *Coprococcus* 1 | rs1519491 | 2 | 22079770 | T | 0.050 | 0.011 | 8.95E-06 |
| Genus *Coprococcus* 1 | rs73167075 | 3 | 165693143 | T | 0.057 | 0.013 | 8.57E-06 |
| Genus *Coprococcus* 1 | rs56405618 | 4 | 174468316 | A | -0.090 | 0.019 | 1.57E-06 |
| Genus *Coprococcus* 1 | rs1576241 | 6 | 72529277 | A | -0.051 | 0.011 | 3.33E-06 |
| Genus *Coprococcus* 1 | rs1762123 | 6 | 150288094 | C | -0.089 | 0.020 | 8.01E-06 |
| Genus *Coprococcus* 1 | rs7784490 | 7 | 132556835 | C | -0.052 | 0.011 | 4.55E-06 |
| Genus *Coprococcus* 1 | rs946513 | 10 | 15913820 | C | 0.206 | 0.046 | 8.62E-06 |
| Genus *Coprococcus* 1 | rs12794898 | 11 | 124554061 | G | 0.090 | 0.020 | 4.92E-06 |
| Genus *Coprococcus* 1 | rs73031725 | 11 | 134622282 | T | 0.168 | 0.036 | 1.98E-06 |
| Genus *Coprococcus* 1 | rs12886051 | 14 | 29898456 | G | -0.052 | 0.012 | 8.01E-06 |
| Genus *Coprococcus* 1 | rs2907920 | 19 | 2600717 | A | 0.056 | 0.013 | 7.65E-06 |
| Genus *Coprococcus* 1 | rs4277593 | 20 | 4319420 | G | -0.059 | 0.011 | 1.14E-07 |
| Genus *Eisenbergiella* | rs12710729 | 2 | 19973210 | C | 0.089 | 0.020 | 9.84E-06 |
| Genus *Eisenbergiella* | rs1553971 | 3 | 111419486 | T | 0.121 | 0.026 | 5.27E-06 |
| Genus *Eisenbergiella* | rs11938607 | 4 | 189060431 | T | 0.098 | 0.022 | 8.22E-06 |
| Genus *Eisenbergiella* | rs13258851 | 8 | 55330814 | A | 0.137 | 0.030 | 7.75E-06 |
| Genus *Eisenbergiella* | rs3812426 | 8 | 50822676 | G | 0.106 | 0.022 | 2.72E-06 |
| Genus *Eisenbergiella* | rs12257723 | 10 | 109417101 | A | -0.095 | 0.021 | 8.85E-06 |
| Genus *Eisenbergiella* | rs11027642 | 11 | 24001840 | C | 0.129 | 0.028 | 4.92E-06 |
| Genus *Eisenbergiella* | rs12278566 | 11 | 124764527 | T | -0.121 | 0.025 | 1.65E-06 |
| Genus *Eisenbergiella* | rs1508033 | 15 | 53375201 | A | 0.092 | 0.020 | 3.23E-06 |
| Genus *Eisenbergiella* | rs2683098 | 15 | 36178565 | C | 0.107 | 0.023 | 2.24E-06 |
| Genus *Eisenbergiella* | rs11079158 | 17 | 53367641 | T | 0.101 | 0.023 | 7.35E-06 |
| Genus *Eisenbergiella* | rs4462860 | 21 | 21610254 | G | 0.094 | 0.020 | 4.16E-06 |
| Genus *Flavonifractor* | rs11811696 | 1 | 237351354 | T | -0.116 | 0.024 | 2.07E-06 |
| Genus *Flavonifractor* | rs12030302 | 1 | 77887835 | A | -0.069 | 0.014 | 5.61E-07 |
| Genus *Flavonifractor* | rs12038887 | 1 | 102287083 | C | 0.094 | 0.021 | 9.37E-06 |
| Genus *Flavonifractor* | rs4949766 | 1 | 77569489 | T | -0.069 | 0.015 | 4.83E-06 |
| Genus *Flavonifractor* | rs6761463 | 2 | 50201547 | G | -0.083 | 0.018 | 8.11E-06 |
| Genus *Flavonifractor* | rs114873521 | 5 | 168218403 | C | -0.130 | 0.029 | 7.13E-06 |
| Genus *Flavonifractor* | rs806808 | 10 | 32381186 | T | 0.067 | 0.014 | 1.18E-06 |
| Genus *Flavonifractor* | rs34066017 | 11 | 44849689 | A | 0.076 | 0.016 | 1.52E-06 |
| Genus *Flavonifractor* | rs11642826 | 16 | 6696929 | G | 0.147 | 0.033 | 6.65E-06 |
| Genus *Flavonifractor* | rs798674 | 16 | 96987 | G | 0.064 | 0.014 | 7.02E-06 |
| Genus *Prevotella* 9 | rs2495052 | 1 | 14161251 | A | 0.084 | 0.019 | 8.97E-06 |
| Genus *Prevotella* 9 | rs9428102 | 1 | 118852817 | A | -0.078 | 0.018 | 4.62E-06 |
| Genus *Prevotella* 9 | rs11685699 | 2 | 11232685 | C | -0.141 | 0.030 | 2.03E-06 |
| Genus *Prevotella* 9 | rs72815774 | 2 | 91954502 | T | -0.176 | 0.039 | 8.78E-06 |
| Genus *Prevotella* 9 | rs12648235 | 4 | 161115744 | T | 0.079 | 0.018 | 7.39E-06 |
| Genus *Prevotella* 9 | rs1304512 | 5 | 63736025 | G | 0.076 | 0.017 | 5.29E-06 |
| Genus *Prevotella* 9 | rs2683313 | 8 | 19115604 | A | -0.072 | 0.015 | 1.69E-06 |
| Genus *Prevotella* 9 | rs10512344 | 9 | 109009495 | C | 0.247 | 0.054 | 3.19E-06 |
| Genus *Prevotella* 9 | rs11199734 | 10 | 122819400 | A | 0.077 | 0.017 | 7.00E-06 |
| Genus *Prevotella* 9 | rs2104588 | 10 | 12497581 | T | 0.106 | 0.024 | 8.13E-06 |
| Genus *Prevotella* 9 | rs7976209 | 12 | 1794470 | T | -0.087 | 0.020 | 7.28E-06 |
| Genus *Prevotella* 9 | rs16966465 | 15 | 38513730 | G | 0.074 | 0.017 | 9.33E-06 |
| Genus *Prevotella* 9 | rs4968431 | 17 | 59421778 | G | 0.064 | 0.014 | 8.58E-06 |
| Genus *Prevotella* 9 | rs7232121 | 18 | 19993452 | G | 0.067 | 0.014 | 3.76E-06 |
| Genus *Prevotella* 9 | rs7237249 | 18 | 71992502 | C | -0.082 | 0.018 | 8.93E-06 |
| Genus *Prevotella* 9 | rs111509883 | 19 | 639161 | T | 0.171 | 0.035 | 1.24E-06 |
| Genus *Prevotella* 9 | rs746764 | 20 | 21470713 | T | -0.092 | 0.019 | 2.04E-06 |
| Genus *Prevotella* 9 | rs117271932 | 22 | 44236460 | A | 0.208 | 0.044 | 2.82E-06 |
| Genus *Prevotella* 9 | rs4821647 | 22 | 37758730 | G | 0.064 | 0.014 | 9.95E-06 |
| Genus *Prevotella* 9 | rs9613013 | 22 | 26152738 | G | 0.092 | 0.020 | 6.10E-06 |
| Genus *Ruminiclostridium* 6 | rs10829821 | 10 | 132651293 | T | -0.098 | 0.022 | 3.47E-06 |
| Genus *Ruminiclostridium* 6 | rs116969552 | 10 | 128214403 | A | -0.167 | 0.038 | 9.16E-06 |
| Genus *Ruminiclostridium* 6 | rs11992182 | 8 | 79766499 | A | 0.063 | 0.014 | 4.65E-06 |
| Genus *Ruminiclostridium* 6 | rs1756364 | 14 | 20954661 | G | 0.100 | 0.020 | 2.54E-07 |
| Genus *Ruminiclostridium* 6 | rs1871858 | 15 | 61047117 | C | -0.105 | 0.024 | 9.12E-06 |
| Genus *Ruminiclostridium* 6 | rs2548459 | 19 | 49209339 | C | 0.055 | 0.012 | 6.40E-06 |
| Genus *Ruminiclostridium* 6 | rs35362464 | 4 | 36478011 | C | 0.072 | 0.017 | 8.99E-06 |
| Genus *Ruminiclostridium* 6 | rs61060922 | 16 | 72136154 | T | 0.159 | 0.032 | 1.09E-06 |
| Genus *Ruminiclostridium* 6 | rs663262 | 11 | 86179076 | T | -0.135 | 0.031 | 3.39E-06 |
| Genus *Ruminiclostridium* 6 | rs67479537 | 19 | 10115515 | T | 0.119 | 0.026 | 9.30E-06 |
| Genus *Ruminiclostridium* 6 | rs71414120 | 14 | 56938952 | T | 0.201 | 0.041 | 1.08E-06 |
| Genus *Ruminiclostridium* 6 | rs72991535 | 18 | 76018244 | T | 0.136 | 0.030 | 4.95E-06 |
| Genus *Ruminiclostridium* 6 | rs73176030 | 7 | 101271282 | T | 0.059 | 0.013 | 7.29E-06 |
| Genus *Ruminiclostridium* 6 | rs77193512 | 11 | 40289063 | A | 0.074 | 0.015 | 1.30E-06 |
| Genus *Ruminiclostridium* 6 | rs792058 | 2 | 5548605 | G | 0.055 | 0.013 | 8.58E-06 |
| Genus *Ruminiclostridium* 6 | rs79968172 | 1 | 240503826 | G | 0.116 | 0.024 | 1.66E-06 |
| Genus *Ruminiclostridium* 6 | rs9555756 | 13 | 111703249 | A | -0.080 | 0.018 | 7.10E-06 |

^1^ Chr, chromosome; IBS, irritable bowel syndrome; SE, standard error; SNP, single nucleotide polymorphism.
